# Do holes in long-lasting insecticidal nets compromise their efficacy against pyrethroid resistant *Anopheles* *gambiae* and *Culex* *quinquefasciatus*? Results from a release–recapture study in experimental huts

**DOI:** 10.1186/s12936-015-0836-7

**Published:** 2015-08-28

**Authors:** Sanjiarizaha Randriamaherijaona, Olivier J T Briët, Sébastien Boyer, Aziz Bouraima, Raphael N’Guessan, Christophe Rogier, Vincent Corbel

**Affiliations:** Institut Pasteur de Madagascar, Antananarivo, Madagascar; Department of Epidemiology and Public Health, Swiss Tropical and Public Health Institute, Basel, Switzerland; University of Basel, Basel, Switzerland; Centre de Recherches Entomologiques de Cotonou (CREC), 06 BP 2604, Cotonou, Republic of Benin; Department of Disease Control, London School of Hygiene and Tropical Medicine, Keppel street, London, WC1E 7HT UK; Unité de Recherche sur les Maladies Infectieuses et Tropicales Emergentes, UM 63, CNRS 7278, IRD 198, INSERM 1095, Aix Marseille Université, Marseille, France; Institut de Recherche Biomédicale des Armées, Brétigny sur Orge, France; Institut de Recherche pour le Développement, Maladies Infectieuses et Vecteurs, Ecologie, Génétique, Evolution et Contrôle (IRD 224-CNRS 5290 UM1-UM2), Montpellier, France; Department of Entomology, Faculty of Agriculture at Kamphaeng Saen, Kamphaeng Saen Campus, Kasetsart University, Nakhon Pathom, 73140 Thailand

**Keywords:** Pyrethroid resistance, *Anopheles* *gambiae*, *Culex* *quinquefasciatus*, LLIN integrity, Mosquito feeding, Mosquito mortality, Malaria transmission

## Abstract

**Background:**

Resistance of malaria vectors to pyrethroids threatens the effectiveness of long-lasting insecticidal nets (LLINs) as a tool for malaria control. Recent experimental hut and observational studies in Benin show that pyrethroid resistance reduces the insecticidal effect and personal protection of LLINs especially when they become torn. The World Health Organization has proposed a threshold for when nets are “too torn” at 1,000 cm^2^ for rectangular holes and 790 cm^2^ for round holes. This study examines whether there is a threshold above which LLINs no longer reduce malaria transmission.

**Methods:**

Intact and artificially-holed LLINs under three months old and untreated nets were tested by releasing mosquitoes from a susceptible *Anopheles* *gambiae* colony, a pyrethroid-resistant *An.* *gambiae* population and a resistant *Culex* *quinquefasciatus* population in closed experimental huts in Southern Benin, West Africa. The efficacy of LLINs and untreated nets was evaluated in terms of protection against blood feeding, insecticidal effect and potential effect on malaria transmission.

**Results:**

Personal protection by both LLINs and untreated nets decreased exponentially with increasing holed surface area, without evidence for a specific threshold beyond which LLINs could be considered as ineffective. The insecticidal effect of LLINs was lower in resistant mosquitoes than in susceptible mosquitoes, but holed surface area had little or no impact on the insecticidal effect of LLINs. LLINs with 22,500 cm^2^ holed surface area and target insecticide content provided a personal protection of 0.60 (95 % CI 0.44–0.73) and a low insecticidal effect of 0.20 (95 % CI 0.12–0.30) against resistant *An. gambiae*. Nevertheless, mathematical models suggested that if 80 % of the population uses such nets, they could still prevent 94 % (95 % CI 89–97 %) of transmission by pyrethroid-resistant *An.* *gambiae*.

**Conclusions:**

Even though personal protection by LLINs against feeding mosquitoes is strongly reduced by holes, the insecticidal effect of LLINs is independent of the holed surface area, but strongly dependent on insecticide resistance. Badly torn nets that still contain insecticide have potential to reduce malaria transmission. The relationship between LLIN integrity and efficacy needs to be understood in order to guide LLIN distribution policy.

**Electronic supplementary material:**

The online version of this article (doi:10.1186/s12936-015-0836-7) contains supplementary material, which is available to authorized users.

## Background

Malaria remains a serious public health problem in Africa with most deaths occurring in children under 5 years old. It is estimated that 207 million cases (95 % confidence interval (CI) 135–287 million) and 627,000 malaria deaths (95 % CI 473,000–789,000) occurred in 2012, of these, 90 % occurred in sub-Saharan Africa [1]. Between 2000 and 2012, the estimated number of malaria cases per 1,000 persons at risk of malaria was reduced by 29 % globally and by 31 % in the African region. This has mostly been credited to the distribution of long-lasting insecticidal nets (LLINs), with 443 million nets distributed over 2010–2014 in sub-Saharan Africa, and implementation of indoor spraying of houses with residual insecticides (IRS) [1]. These two vector control methods have become essential components of worldwide malaria control and elimination efforts [2, 3]. Unfortunately, resistance to pyrethroids has spread dramatically in major malaria vectors in Africa, hence representing a serious threat for the success of African national malaria control programmes (NMCPs) relying on LLINs and IRS [4]. In addition, pyrethroid resistance is strong in *Culex* *quinquefasciatus,* a filariasis vector and major urban pest in most of African countries [5, 6]. The biting nuisance caused by *Cx.* *quinquefasciatus* and other non-malaria vectors can have severe implications for malaria control because a positive correlation exists between the biting nuisance and the effective use of LLINs by populations [7, 8].

While untreated nets in good condition can protect against malaria transmission, worn untreated nets do not protect [9]. Port and Boreham [10] found that the number of mosquitoes biting with untreated nets is directly related to the net condition (which they expressed as the sum of the round hole circumferences and the length of the splits). The strong knock down (KD) and excito-repellent effects of pyrethroids allow insecticide treated nets (ITNs) to remain effective even when they become holed [11–13]. However, the impact of holed ITNs and LLINs against pyrethroid-resistant mosquitoes is not yet elucidated. Irish and colleagues [14] demonstrated that the efficacy of ITNs decreases against resistant *Cx.* *quinquefasciatus* mosquitoes as nets become increasingly holed. This finding was reinforced recently by an observational study conducted at household level in Benin that showed little or no protection by ITNs when the mosquitoes are resistant and when the nets are dirty and holed [15].

As part of World Health Organization Pesticide Evaluation Scheme (WHOPES) requirement for LLIN evaluation, nets must be washed and deliberately holed to simulate field conditions of LLIN use [16]. Although World Health Organization (WHO) guidelines provide clear thresholds for bioefficacy indicators (mortality ≥ 80 % and/or KD60 ≥ 95 %), no threshold has been established for net integrity. Under operational conditions, the physical integrity of nets is evaluated by recording the number and the size of holes in the different panels of the nets. Recently, the WHO proposed a hole index for the monitoring of LLIN integrity in the field [16]. This index has been used to standardize damage assessment in LLINs and to categorize them as “effective nets” (offering substantial protection against mosquito bites) or as “ineffective nets” (offering little or no protection against mosquito bites) [17], although no official threshold of holes index exist above which an LLIN would lose its efficacy. The WHO vector control group committee recently proposed to classify a net with a total hole surface area of more than 1,000 cm^2^ for rectangular and 790 cm^2^ for circular holes as too torn “where its protective efficacy for the user is in serious doubt and the net should be replaced as soon as possible” [18]. However, these proposed thresholds have little evidence to support them.

Here, the impact of physical damage on personal protection and insecticidal effect of a WHOPES recommended LLIN (PermaNet 2.0) and an untreated net was investigated against susceptible and pyrethroid-resistant *Anopheles* *gambiae* and *Cx.* *quinquefasciatus* over a range of hole sizes and numbers. Mathematical models [19, 20] were then used to predict the community protection, overall insecticidal effect and effect on transmission that holed and intact LLINs would confer in a population where 40, 60 or 80 % of the people used the nets.

## Methods

### Mosquitoes

The pyrethroid-susceptible KISUMU strain of *An.* *gambiae* sensu stricto from Kenya [21] was used for bioassays and experimental hut trials. This reference strain has been maintained at the insectary of the Entomological Research Centre of Cotonou (CREC), Cotonou, Benin for more than 15 years. Samples from two wild local populations of *An.* *gambiae* and *Cx.* *quinquefasciatus* were used in the experimental huts trial: *An.* *gambiae* sensu lato (hereafter: *An.* *gambiae* AKRON) was collected at larval stage in Akron (6°30′N; 2°47′E) in the district of Porto-Novo, the capital of Benin (coastal Guinean area) and contains a high frequency of the 1014F mutation and enhanced P450 activity [22, 23]. *Culex* *quinquefasciatus* larvae were collected in Cotonou and present high level of pyrethroid resistance due to *kdr* mutation and enhanced metabolic detoxification [6]. Larvae were raised to adults in the insectary of CREC before use in the tests.

### Insecticide susceptibility test

Adult females of *An.* *gambiae* AKRON and *Cx.* *quinquefasciatus* reared from larval collections were tested alongside *An.* *gambiae* KISUMU in bioassays to confirm their insecticide resistance status prior the release in experimental huts. One hundred unfed 3–4 days old females from each population were exposed for 1 h to filter papers treated with a standard diagnostic dose of 0.05 % deltamethrin using WHO test kits [24]. Following exposure, mosquitoes were transferred into holding tubes and provided with cotton pads wetted with a 10 % honey solution. Approximately 25 mosquitoes from each population were exposed to untreated filter papers to serve as controls. Mortality was recorded after 24 h.

### WHO cone test

Prior to release-recapture experiments in huts, WHO cone tests were used to check the bioefficacy of the experimental nets on *An.* *gambiae* KISUMU in terms of knock down (KD) and lethality. These bioassays were conducted on the 16 intact nets immediately after they had been unwrapped. Mosquitoes were released in a cone fitted with PermaNet 2.0 netting or untreated netting (negative control) for 3 min exposure [16]. Fifty non-blood fed, 2- to 5-day old susceptible *An.* *gambiae* mosquitoes were tested on each net (five mosquitoes per cone × two replicates × five locations). Bioassays were carried out at 27 °C ± 2 % and 75 ± 10 % relative humidity. KD was measured 60 min after exposure and mortality was measured after 24 h.

### Experimental huts

Seven West African style experimental huts with veranda traps [16], originally used in experiments that allowed mosquitoes to freely enter, were modified for these experiments. Modifications included closing the eaves and window slits to prevent wild mosquitoes from entering and released mosquitoes from escaping.

### Human subjects and experimental procedure

Seven adult men were recruited for the study from the local community to sleep in the seven huts. Before participating in the study, sleepers were informed about the study objective and then signed an informed consent form. Sleepers were instructed not to kill mosquitoes in the hut, and were provided with free anti-malarial drugs if testing positive for malaria. Every test night, each sleeper entered a hut (one sleeper per hut) at 09.00 PM and remained inside until dawn (06:00 AM). Sleepers were provided with water for drinking and a urinal receptacle. About fifty, 4–5 day old female mosquitoes were introduced in a hut 15 min after the sleepers came in. At 06:00 AM, sleepers woke up and collected the alive and dead mosquitoes present under their net (if any) using aspirators and then left the nets to close the curtain of the veranda. They subsequently collected mosquitoes from the floor, walls and roof of the hut, and finally from the veranda trap. The location (in the hut i.e. the area outside the bed net exclusive of the veranda, in the veranda or under the net) of mosquitoes and their physiological status (dead or alive and fed or unfed) were recorded. The surviving mosquitoes were placed in small cups and provided with 10 % honey solution for 24 h to assess delayed mortality.

### Bed net preparation and study design

The LLINs used in the tests were PermaNet 2.0 (Vestergaard Frandsen S.A.), a WHO recommended LLIN made of 100 denier polyester netting (190 cm × 180 cm × 150 cm) containing a target concentration of 55 mg/m^2^ of deltamethrin [25]. Untreated nets made from 100-denier polyester netting (190 cm × 200 cm × 200 cm) served as control nets. Nets were first unpacked in the laboratory for bioassays. After the assays, holes were cut in the nets. Each panel of experimental nets had one or more circular holes cut into it with scissors. Hole sizes were 3, 30 and 300 cm^2^ and these were crossed in a full factorial design with the net types (LLIN and untreated) and the following number of holes:

- 5 holes, one in the centre of each panel of the net
- 10 holes, two on a diagonal in each panel of the net, configured as on a playing die
- 25 holes, five in each panel of the net, configured as on a playing die
- 45 holes, nine in each panel of the net, configured in a three by three grid
- 75 holes, fifteen in each panel of the net, configured in a three rows by five columns grid.

The hole position for each hole number per panel is provided in Additional file 1. In addition, both intact LLINs and untreated nets were tested (Table 1). Nets were hung inside the huts 2–3 days after unpacking. Each test night, all arms with nets with holes had holes of the same size (but differed in the number of holes, with hut numbers 2, 3, 4, 5 and 6 containing nets with 5, 10, 25, 45 and 75 holes, respectively). Every night, one experimental hut (hut number 1) without a net was used as control to check the background mortality and blood feeding success of the released female mosquitoes, and one hut (hut number 7) contained an intact net.

**Table 1:** Experiment arms tested for each mosquito species

Type	Hole size (cm^2^)	Hole no.	Holed surface area (cm^2^)	Holed surface area (%)	Condition^a^	Insecticide
Untreated net	0	0	0	0.000	G	NA
Untreated net	3	5	15	0.008	G	NA
Untreated net	3	10	30	0.016	G	NA
Untreated net	3	25	75	0.039	G	NA
Untreated net	3	45	135	0.070	D	NA
Untreated net	3	75	225	0.117	D	NA
Untreated net	30	5	150	0.078	D	NA
Untreated net	30	10	300	0.156	D	NA
Untreated net	30	25	750	0.391	D	NA
Untreated net	30	45	1,350	0.703	T	NA
Untreated net	30	75	2,250	1.172	T	NA
Untreated net	300	5	1,500	0.781	T	NA
Untreated net	300	10	3,000	1.563	T	NA
Untreated net	300	25	7,500	3.906	T	NA
Untreated net	300	45	13,500	7.031	T	NA
Untreated net	300	75	22,500	11.719	T	NA
No net	Inf	NA	Inf	100.000	NA	NA
PermaNet 2.0	0	0	0	0.000	G	Deltamethrin
PermaNet 2.0	3	5	15	0.010	G	Deltamethrin
PermaNet 2.0	3	10	30	0.021	G	Deltamethrin
PermaNet 2.0	3	25	75	0.052	G	Deltamethrin
PermaNet 2.0	3	45	135	0.093	D	Deltamethrin
PermaNet 2.0	3	75	225	0.155	D	Deltamethrin
PermaNet 2.0	30	5	150	0.103	D	Deltamethrin
PermaNet 2.0	30	10	300	0.207	D	Deltamethrin
PermaNet 2.0	30	25	750	0.517	D	Deltamethrin
PermaNet 2.0	30	45	1,350	0.930	T	Deltamethrin
PermaNet 2.0	30	75	2,250	1.550	T	Deltamethrin
PermaNet 2.0	300	5	1,500	1.033	T	Deltamethrin
PermaNet 2.0	300	10	3,000	2.066	T	Deltamethrin
PermaNet 2.0	300	25	7,500	5.165	T	Deltamethrin
PermaNet 2.0	300	45	13,500	9.298	T	Deltamethrin
PermaNet 2.0	300	75	22,500	15.496	T	Deltamethrin

^a^Classification proposed by WHO: *G* good (holed surface area < 80 cm^2^), *D* damaged (80 < holed surface area < 790), *T* torn (holed surface area > 790 cm^2^).

Sleepers were rotated between huts on successive nights to avoid bias in attractiveness to mosquitoes. First arms with untreated nets were tested during three nights and replicated once (during three nights), before arms with LLINs were tested (during three nights) and replicated once (during three nights). After every release-recapture experiment night, huts were thoroughly washed and aired to prevent any insecticide carry over.

Experiments with *An.* *gambiae* KISUMU were performed over 12 nights between 14 February and 18 March 2012, experiments with *An.* *gambiae* AKRON were performed between 10 and 21 April and experiments with *Cx.* *quinquefasciatus* were performed between 22 April and 3 May 2012. Over the period 14 February–3 May 2012, the minimum daily temperature at Cotounou airport [26] varied between 22 and 29 °C, the maximum daily temperature varied between 29.5 and 37 °C, the minimum daily relative humidity varied between 20 and 80 %, and the maximum daily relative humidity varied between 84 and 100 %. At the end of the experiment, nets were unwashed and less than three months old.

### Analysis of net performance

Data were entered in standardized data entry sheets in Microsoft Excel (see Additional file 2) by trained staff on a daily basis, stored on the computer of the principal investigator (VC) and transferred to R software (v2.14) for further analysis. Fisher’s central exact test was performed with the function ‘fisher.exact’ of the ‘exact2x2’ R package.

The effect of holes in the nets and insecticide in the LLINs was analysed by performing regression analysis on crude experiment outcomes of the proportion of fed mosquitoes, the proportion of dead mosquitoes, the proportion in the veranda and the proportion found under the net. The data from the experiment arm without nets were excluded from this analysis. Regressions were performed using a generalized linear model of the binomial family with logit link function with data from replicates aggregated by experiment arm (Table 1). Initial models considered the type of net (untreated or LLIN) and the mosquito species as two explanatory variables. Further models also considered the logarithmically transformed holed surface area in cm^2^ augmented with one (to avoid problems with taking the log of zero with intact nets) and the holes size as a categorical variable. Regression models were selected with the R function ‘stepAIC’ using forward and backward stepwise comparison of models with all possible variable interactions based on the Akaike’s information criterion (AIC). A likelihood ratio test with a type I error size alpha of 0.05 was used to compare more complex models with models with fewer explanatory variables.

Risk ratio analysis was done to test whether the proportions of dead and fed mosquitoes found under the net and elsewhere in the huts varied with the holed surface area.

Using mathematical models, the following outcome measures of protective effects of bed nets were estimated for each treatment arm: protection against feeding, community protection against feeding, overall insecticidal effect (of an individual treated hut), community insecticidal effectiveness and protection against transmission (see "Appendix"). For this analysis, for each treatment arm and control (no net), mosquitoes were aggregated over the entire experiment into the following four categories: unfed alive, unfed dead, fed alive and fed dead. In order to calculate confidence intervals around the estimates of the outcome measures for each treatment, the numbers of mosquitoes in the four categories were presumed to be multinomially-distributed, and the outcome measure was calculated for 30,000 samples drawn from the distribution. In order to calculate the outcome measures, estimates of deterrence from the huts (i.e. reduction in entry rates) were required. Since this parameter was not available in this assay (mosquitoes were released inside huts), deterrence of each mosquito species was estimated from data in literature. For *An.* *gambiae* KISUMU, data collected by Tungu and colleagues [27] on a susceptible [28] *An.* *gambiae* population in Zeneti, Tanzania, were used. Tungu and colleagues collected a total of 723 mosquitoes inside huts with control nets and 574 mosquitoes in huts with unwashed PermaNet 2.0 nets. For the pyrethroid resistant *An.* *gambiae*, data collected by N’Guessan and colleagues [29, 30] were used. They collected 185 mosquitoes inside huts with control nets, and 114 mosquitoes in huts with unwashed PermaNet 2.0 nets. For *Cx.* *quinquefasciatus*, data collected by Irish and colleagues [14] in Ladji (Cotonou), Benin, were used. They collected 688 mosquitoes inside huts with control nets, and 577 mosquitoes in huts with alpha-cypermethrin treated net (40 mg/m^2^). Even though alpha-cypermethrin is a different insecticide from deltamethrin, Mosha and colleagues [31] found no difference in deterrence from hut entry between alpha-cypermethrin and deltamethrin of *Cx.* *quinquefasciatus* and *Anopheles* *arabiensis* in Tanzania. For the calculation of community level outcome measures, it was presumed that in intervention arms, 40, 60 or 80 % of the population had access to and used a net. For the calculation of predicted effectiveness on transmission, the mathematical model by Chitnis and colleagues [32] was used with the default parameter values published by Chitnis and colleagues [32] for settings without alternative hosts (e.g. cattle) present, except where treatment arm specific parameterization was taken from the data. The probability of *Plasmodium* transmission from humans to susceptible mosquitoes was taken as 0.03 per bite for both the population using a net and the population without access to a net. All analyses were performed in R statistical software, with calls to the JAGS software package [33].

### Ethical statement

WHOPES ethical procedures for phase II trials approved by the WHO ethical review board were followed. The participation of the sleepers in the trial did not enhance their risk of malaria as all released mosquitoes were raised from larvae and had never fed on blood prior to release. The study was conducted in the framework of the evaluation of insecticidal products in experimental huts, for which ethical approval was obtained directly from the Benin Ministry of Health (Autorisation administrative de recherche No 1702/MS/DC/SGM/DRS/SRAO/SA of 31 March 2008). The study was conducted before the establishment of the first ethical review board in 2013 in Benin.

## Results

### Insecticide susceptibility status

Results from the WHO susceptibility test showed a mortality rate of 37 % (n = 100, 95 % CI 28–47) and 20 % (n = 100, 95 % CI 13–29) after exposure to 0.05 % deltamethrin in *An.* *gambiae* AKRON and *Cx.* *quinquefasciatus*, respectively. The mortality rate was 100 % (n = 100, 95 % CI 96–100) with the susceptible strain *An.* *gambiae* KISUMU. In control batches, mortality was low with 3.8 % (n = 26, 95 % CI 0–20) dead in *An.* *gambiae* AKRON, 0 % (n = 25, 95 % CI 0–14) dead in *Cx.* *quinquefasciatus*, and 4 % (n = 25, 95 % CI 0–20) dead in *An.* *gambiae* KISUMU.

### WHO cone test

WHO cone bioassays showed that mortality rate of *An.* *gambiae* KISUMU was 0.87 % (n = 800, 95 % CI 0.35–1.79) with untreated nets and 100 % with PermaNet 2.0 (n = 800, 95 % CI 99.5–100). KD after 60 min was 0.25 % (95 % CI 0.03–0.90) with untreated nets and 99.25 % (95 % CI 98.37–99.72) with PermaNet 2.0.

### Mosquito release-recapture assay in huts: crude experiment outcomes

**Fig. 1:**
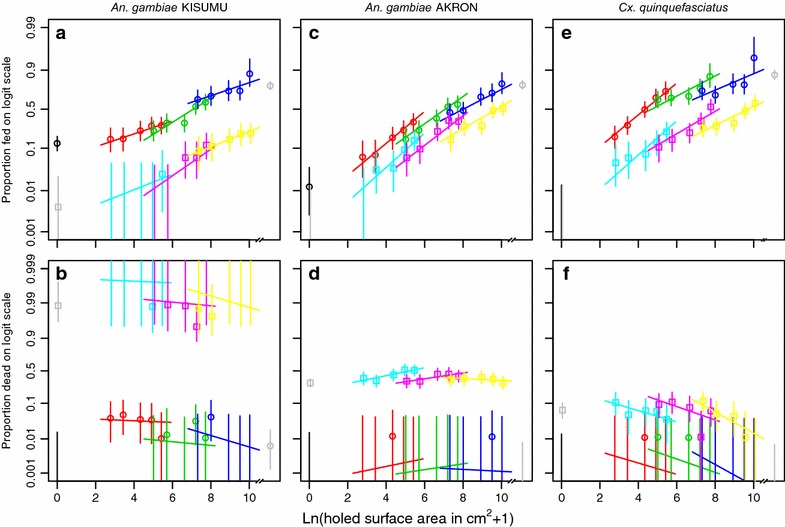
Proportions of mosquitoes fed and dead depending on the holed surface area on three mosquito species. The *first column of panels* **a**, **b** is for *An.* *gambiae* KISUMU strain, the *second column of panels* **c**, **d** is for *An.* *gambiae* collected in Akron, and the third row of panels **e**, **f** is for *Cx.* *quinquefasciatus* collected in Cotonou. The first row of panels **a**, **c**, **e** shows the proportion fed, the *second row of panels* **b**, **d**, **f** shows the proportion dead. *Symbols* show mean values with *grey squares* representing intact LLINs; *cyan squares* LLINs with holes of 3 cm^2^; *magenta squares* LLINs with holes of 30 cm^2^; *yellow squares* LLINs with holes of 300 cm^2^; *black circles* intact untreated nets; *red circles* untreated nets with holes of 3 cm^2^; *lime green* *circles* untreated nets with holes of 30 cm^2^; *dark blue circles* untreated nets with holes of 300 cm^2^. *Grey circles* represent the results from huts without nets. *Vertical lines* show 95 % confidence intervals, and (*non-vertical lines*) show the fitted relationships from logistic regressions.

**Fig. 2:**
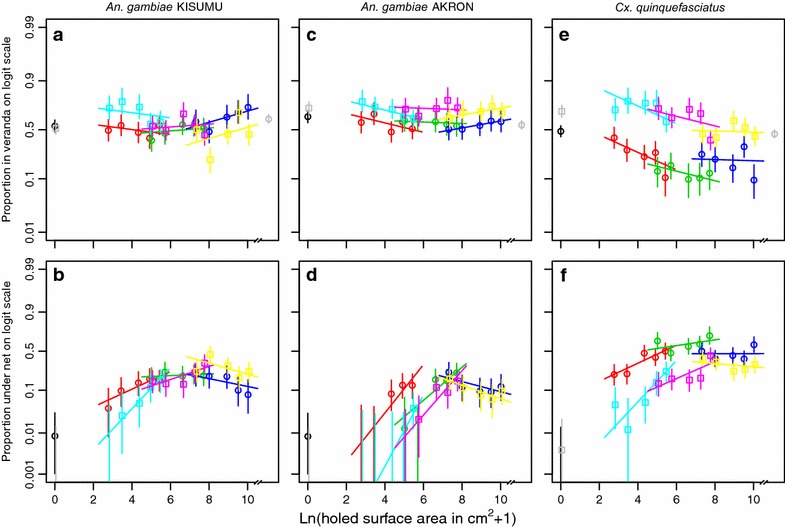
Proportions of mosquitoes in the veranda and under the net depending on the holed surface area for three mosquito species. The *first column of panels* **a**, **b** is for *An.* *gambiae* KISUMU strain, the *second column of panels* **c**, **d** is for *An.* *gambiae* collected in Akron, and the *third column of panels* **e**, **f** is for *Cx.* *quinquefasciatus* collected in Cotonou. The *first row of panels* **a**, **c**, **e** shows the proportion found in the veranda trap, the *second row of panels* **b**, **d**, **f** shows the proportion found under the net. Legend further as in Fig. 1.

The results for the crude outcomes are shown in Figs. 1 and 2 and in Tables 2, 3 and 4. The proportion of fed mosquitoes ranged from 0 to 0.95 depending on the treatment and mosquito species. Without a net, the proportion feeding was 0.79 for *An.* *gambiae* KISUMU, 0.80 for *An.* *gambiae* AKRON, and 0.87 for *Cx.* *quinquefasciatus*. The proportion fed was lower with LLINs compared to untreated nets, irrespective of the mosquito species. Compared to the proportion fed with an intact LLIN, the proportion fed was significantly different (Fisher’s exact test, alpha = 0.05) with LLINs with 750 cm^2^ or more, with 30 cm^2^ or more and with 225 cm^2^ or more holed surface area for *An.* *gambiae* KISUMU, *An.* *gambiae* AKRON, and *Cx.* *quinquefasciatus*, respectively. For *An.* *gambiae* AKRON and *Cx.* *quinquefasciatus*, the difference in feeding with LLINs compared to untreated nets was smaller than for *An.* *gambiae* KISUMU. The proportion of fed mosquitoes on a logit scale had an approximately linear relationship with the logarithmically transformed holed surface area (see Fig. 1 panels a, c, and e). The regression model with two explanatory variables (mosquito species and type of net) explained only 34.5 % of the total variation in the data (Table 5; Additional file 3). Addition of the logarithmically transformed holed surface area increased (likelihood ratio test, p ≤ 0.05) the amount of explained variation by 58.3 percentage points from 34.5 % to a total of 92.8 %. Further addition of the hole size variable (to the model with mosquito species, type of net, and holed surface area) increased the amount of variation in the data that was explained (likelihood ratio test, p ≤ 0.05) by 5.1 percentage points from 92.8 to 97.9 %. The regression model with three explanatory variables predicted that the odds of feeding by *An.* *gambiae* KISUMU increased by 1.52 for every unit increase in the logarithmically transformed holed surface area (e.g. if the surface area increases from 0 to 1.72 cm^2^, or from 100 to 273.5 cm^2^) in untreated nets and LLINs. For *An.* *gambiae* AKRON and *Cx.* *quinquefasciatus*, these multiplication factors were 1.70 and 1.65, respectively. Again using the three explanatory variable regression model, the threshold for the holed surface area in untreated nets at which the proportion of blood fed mosquitoes would be the same as that in the experiment arm without a net was calculated and expressed as a percentage of the total net surface (192,000 cm^2^). This threshold was 15.2 % (95 % CI 7.1–37.3) for *An.* *gambiae* KISUMU, 12.0 % (95 % CI 6.8–23.5) for *An.* *gambiae* AKRON and 3.0 % (95 % CI 1.7–5.7) for *Cx.* *quinquefasciatus*. For LLINs with total net surface of 145,200 cm^2^, these thresholds were over 100 % of the total net surface (Table 6). With intact untreated nets, *An.* *gambiae* AKRON and *Cx.* *quinquefasciatus* were unable to feed, whereas 13 % (95 % CI 9–18) of *An.* *gambiae* KISUMU obtained a blood meal. For *An.* *gambiae* KISUMU, the size of holes had little influence on the feeding success. Interestingly, with the same total holed surface area, more females of *An.* *gambiae* AKRON and *Cx.* *quinquefasciatus* were fed if the holes were smaller (but more numerous) than if the holes were larger in size (but smaller in number).

**Table 2:** Results for *Anopheles* *gambiae* KISUMU

Hole size (cm^2^)	Hole count	Holed surface area (cm^2^)	Untreated net									LLIN								
			n	Dead (%)^a^		Fed (%)^a^		Veranda (%)^a^		Under net (%)^a^		n	Dead (%)^a^		Fed (%)^a^		Veranda (%)^a^		Under net (%)^a^	
0	0	0	236	0.0	a	12.7	a	54.2	c–e	0.8	a	250	98.8	a	0.4	a	51.6	a	0.0	a
3	5	15	77	3.9	b, c	15.6	a, b	49.4	a–e	3.9	a, b	80	100.0	a	0.0	a, b	72.5	c–e	0.0	a, b
3	10	30	82	4.9	c	15.9	a, b	54.9	a–f	9.8	b–d	77	100.0	a	0.0	a, b	77.9	e	2.6	a–c
3	25	75	83	3.6	b, c	22.9	b, c	47.0	a–d	14.5	c–e	79	100.0	a	0.0	a, b	73.4	d, e	5.1	b–d
3	45	135	86	3.5	b, c	27.9	b, c	40.7	a, b	17.4	c–e	78	98.7	a	0.0	a, b	56.4	a–c	11.5	c–e
3	75	225	98	1.0	a–c	28.6	b, c	55.1	b–f	16.3	c–e	80	100.0	a	2.5	a–c	58.8	a–d	16.3	e
30	5	150	78	0.0	a–c	23.1	b, c	38.5	a	17.9	c–e	89	100.0	a	0.0	a, c	53.9	a, b	15.7	e
30	10	300	76	1.3	a–c	31.6	c	46.1	a–c	23.7	e	87	98.9	a	0.0	a–c	49.4	a	13.8	d, e
30	25	750	86	0.0	a–c	31.4	c	55.8	b–f	19.8	c–e	82	98.8	a	6.1	b–d	67.1	b–e	13.4	d, e
30	45	1,350	93	3.2	b, c	53.8	d	53.8	a–f	21.5	d, e	83	95.2	a	6.0	b–d	55.4	a, b	22.9	e–g
30	75	2,250	94	1.1	a–c	59.6	d, e	52.1	a–e	20.2	d, e	86	100.0	a	11.6	d, e	44.2	a	33.7	g, h
300	5	1,500	71	0.0	a–c	63.4	d, e	59.2	c–g	21.1	d, e	70	98.6	a	8.6	c–e	50.0	a	24.3	e–g
300	10	3,000	71	4.2	b, c	67.6	d, e	47.9	a–d	19.7	c–e	82	97.6	a	11.0	c–e	20.7		45.1	h
300	25	7,500	72	0.0	a–c	73.6	e	63.9	d–g	19.4	c–e	91	100.0	a	15.4	d, e	45.1	a	30.8	f–h
300	45	1,3500	80	0.0	a–c	73.8	e	68.8	f, g	10.0	b–d	76	100.0	a	19.7	e	68.4	b–e	18.4	e, f
300	75	22,500	75	0.0	a–c	88.0	f	73.3	g	8.0	b, c	83	100.0	a	20.5	e	44.6	a	24.1	e–g
Inf	NA	Inf	483	0.6	a, b	78.9	e	61.9	e–g											

^a^Percentages with common letters are not significantly different in pairwise comparisons between experiment arms in a column (Fisher exact test, alpha = 0.05).

**Table 3:** Results for *Anopheles* *gambiae* AKRON

Hole size (cm^2^)	Hole count	Holed surface area (cm^2^)	Untreated net									LLIN								
			n	Dead (%)^a^		Fed (%)^a^		Veranda (%)^a^		Under net (%)^a^		n	Dead (%)^a^		Fed (%)^a^		Veranda (%)^a^		Under net (%)^a^	
0	0	0	241	0.0	a	1.2		64.3	b	0.8	a	277	30.7	a	0.0	a	72.6	a, b	0.0	a
3	5	15	79	0.0	a	6.3	a	58.2	a, b	0.0	a	104	37.5	a, b, e	0.0	a, b	77.9	b	0.0	a, b
3	10	30	85	0.0	a	7.1	a	67.1	b	0.0	a	125	33.6	a–c	3.2	b, c	73.6	a, b	0.0	a, b
3	25	75	84	1.2	a	16.7	a,b	47.6	a	8.3	b, c	116	43.1	b–d	3.4	b, c	71.6	a, b	0.0	a, b
3	45	135	85	0.0	a	23.5	b, c	58.8	a, b	12.9	c, d	120	51.7	d	9.2	c, d	64.2	a	0.0	a, b
3	75	225	86	0.0	a	32.6	c, d	51.2	a	12.8	c, d	130	50.8	d, e	14.6	d, e	62.3	a	3.8	b–d
30	5	150	78	0.0	a	15.4	a, b	60.3	a, b	1.3	a, b	99	33.3	a–c	6.1	c, d	71.7	a, b	0.0	a, b
30	10	300	81	0.0	a	23.5	b,c	61.7	a, b	0.0	a	94	33.0	a–c	9.6	c, d	64.9	a, b	2.1	a–c
30	25	750	83	0.0	a	37.3	c,d	59.0	a, b	16.9	c, d	104	44.2	b–d	22.1	e, f	72.1	a, b	11.5	e, f
30	45	1,350	75	0.0	a	53.3	e, g	56.0	a, b	16.0	c, d	90	44.4	b–d	34.4	f, g	78.9	b	8.9	c–f
30	75	2,250	83	0.0	a	56.6	e–g	54.2	a, b	22.9	d	114	41.2	a–d	33.3	f, g	72.8	a, b	15.8	f
300	5	1,500	77	0.0	a	45.5	d, g	50.6	a	23.4	d	92	35.9	a–c	15.2	d, e,h	63.0	a	17.4	f
300	10	3,000	85	0.0	a	48.2	d, g	50.6	a	11.8	c, d	80	37.5	a–d	27.5	f, h	68.8	a, b	11.3	d–f
300	25	7,500	84	0.0	a	66.7	e, f, h	54.8	a, b	9.5	c	104	39.4	a–d	27.9	f	69.2	a, b	7.7	c–f
300	45	13,500	87	1.1	a	71.3	f, h, i	59.8	a, b	9.2	b, c	96	36.5	a–c	47.9	g, i	74.0	a, b	6.3	c–e
300	75	22,500	83	0.0	a	80.7	h, i	59.0	a, b	12.0	c, d	104	30.8	a–c	51.0	i	68.3	a, b	9.6	c–f
Inf	NA	Inf	475	0.0	a	79.6	i	55.4	a, b											

^a^Percentages with common letters are not significantly different in pairwise comparisons between experiment arms in a column (Fisher exact test, alpha = 0.05).

**Table 4:** Results for *Culex* *quinquefasciatus*

Hole size (cm^2^)	Hole count	Holed surface area (cm^2^)	Untreated net									LLIN								
			n	Dead (%)^a^		Fed (%)^a^		Veranda (%)^a^		Under net (%)^a^		n	Dead (%)^a^		Fed (%)^a^		Veranda (%)^a^		Under net (%)^a^	
0	0	0	267	0.0	a	0.0		48.3	h	0.0	a	259	6.6	a, c, d	0.0	0	69.5	d–f	0.4	a
3	5	15	80	0.0	a	17.5	a	41.3	g, h	21.3	b	86	10.5	b, c	4.7	a	70.9	d–f	4.7	b, c
3	10	30	87	0.0	a	28.7	a	28.7	e–g	21.8	b	83	4.8	a, b, d	6.0	a, b	78.3	f	1.2	a, b
3	25	75	91	1.1	a	49.5	b	23.1	c–f	47.3	c–f	95	6.3	a, b	7.4	a, b	76.8	e, f	5.3	b, c
3	45	1,35	87	0.0	a	65.5	c	26.4	d–g	41.4	c, d	82	6.1	a, b	13.4	a–c	76.8	e, f	14.6	c, d
3	75	2,25	86	0.0	a	73.3	c, d	10.5	a, b	50.0	c–g	83	3.6	a, b, d	21.7	c–e	59.0	b–d	24.1	d, e
30	5	1,50	89	1.1	a	62.9	b, c	13.5	a–c	64.0	g, h	86	9.3	b, c	10.5	a, b, d	72.1	d–f	14.0	c, d
30	10	3,00	95	0.0	a	65.3	c	16.8	a–e	47.4	c–f	92	10.9	b, c	15.2	b, c, f	63.0	c–e	17.4	d
30	25	7,50	92	1.1	a	67.4	c, d	9.8	a, b	56.5	d–h	91	7.7	a, b	20.9	c–e	71.4	d–f	16.5	d
30	45	1,350	97	0.0	a	76.3	c, e	10.3	a, b	59.8	f–h	89	1.1	a, d	33.7	e, g	67.4	c–f	18.0	d
30	75	2,250	88	0.0	a	86.4	e–g	12.5	a–c	70.5	h	96	6.3	a, b	53.1	h	38.5	a	43.8	g
300	5	1,500	84	0.0	a	73.8	c, e	25.0	c–f	50.0	c–g	97	11.3	b, c	25.8	c, e, f	45.4	a, b	40.2	f, g
300	10	3,000	90	0.0	a	68.9	c, d	21.1	b–f	40.0	c	97	5.2	a, b, d	27.8	e, f	41.2	a	35.1	e–g
300	25	7,500	91	0.0	a	80.2	d, e, g	15.4	a–d	44.0	c–e	90	4.4	a, b, d	31.1	e	60.0	b–d	24.4	d, e
300	45	13,500	94	0.0	a	79.8	d, e, g	31.9	f, g	39.4	c	91	1.1	a, d	47.3	g, h	52.7	a–c	26.4	d–f
300	75	22,500	95	0.0	a	94.7	f	9.5	a	58.9	e–h	94	0.0	d	58.5	h	42.6	a	31.9	e–g
Inf	NA	Inf	536	0.0	a	87.3	g	45.3	h											

^a^Percentages with common letters are not significantly different in pairwise comparisons between experiment arms in a column (Fisher exact test, alpha = 0.05).

**Table 5:** Results from regression analysis

Outcome	Var.	Model	McFadden’s pseudo R^2^	Difference
Fed	2	Species + Type + Species:Type	0.345	
	3	Species + **logTHSp1** + Type + Species:Type + **Species:logTHSp1**	0.928	0.583*
	4	Species + logTHSp1 + Type + **Size** + Species:Type + **Species:Size** + **logTHSp1:Size** + Species:logTHSp1 + **logTHSp1:Type** + **Species:logTHSp1:Size**	0.979	0.051*
Dead	2	Species + Type + Species:Type	0.979	
	3	Species + **logTHSp1** + Type + Species:Type + **Species:logTHSp1**	0.980	0.001
	4	Species + logTHSp1 + Type + **Size** + Species:Type + Species:logTHSp1 + **Species:Size** + **logTHSp1:Size**	0.989	0.009*
Veranda	2	Species + Type + Species:Type	0.652	
	3	Species + **logTHSp1** + Type + Species:Type + **Species:logTHSp1**	0.762	0.110*
	4	Species + logTHSp1 + Type + **Size** + Species:Type + Species:logTHSp1 + **Type:Size** + **logTHSp1:Size** + **Species:Size** + **Species:Type:Size**	0.887	0.125*
Under net	2	Species + Type + Species:Type	0.398	
	3	Species + **logTHSp1** + Type + Species:Type + **logTHSp1:Type** + **Species:logTHSp1** + ***Species:logTHSp1:Type***	0.739	0.341*
	4	Species + logTHSp1 + Type + **Size** + logTHSp1:Size + Species:Type + logTHSp1:Type + Species:logTHSp1 + **Species:Size** + **Type:Size** + **Species:logTHSp1:Size** + **logTHSp1:Type:Size** + **Species:Type:Size**	0.945	0.206*

Var.: number of explanatory variables considered, with two variables these are Species and Type (of net), with three variables the variable logTHSp1 (log of total holed surface area in cm^2^ plus 1) is added, and with four variables the variable (hole) Size is added; terms in bold face: term added compared to less complex model; terms in italic: term removed in more complex model; two variables separated by ‘:’ indicate an interaction term; differences marked with an asterisk (*) are significantly different (likelihood ratio test, alpha = 0.05).

**Table 6:** Extrapolated holed surface area in cm^2^ at which the proportion feeding is equal to the feeding in the no-net experiment arm

Species	Net	Median	Lower 95 % credible boundary	Upper 95 % credible boundary
*An.* *gambiae* KISUMU	Untreated	29,205 (15.2)	13,716 (7.1)	71,624 (37.3)
	LLIN	1,131,491 (>100)	359,992 (>100)	6,453,106 (>100)
*An.* *gambiae* AKRON	Untreated	23,094 (12.0)	13,118 (6.8)	45,095 (23.5)
	LLIN	235,643 (>100)	111,220 (76.7)	591,737 (>100)
*Cx.* *quinquefasciatus*	Untreated	5,702 (3.0)	3,172 (1.7)	11,035 (5.7)
	LLIN	782,090 (>100)	295,433 (>100)	2,704,911 (>100)

Holed surface area is given in cm^2^ with between parenthesis holed surface area in per cent of the total net surface. The relationship used is that found with the three-variable regression model (Table 5; Additional file 3).

The proportion of dead mosquitoes was higher with LLINs than with untreated nets, irrespective of the species (Fig. 1 panels b, d, and f). The proportion of dead mosquitoes in experiment arms with intact untreated nets was not different from that of the experiment arms with no net (Tables 2, 3 and 4). The proportion of dead mosquitoes ranged from 0 to 1 for *An.* *gambiae* KISUMU. For *An.* *gambiae* AKRON, the proportion dead ranged from 0 to 0.51. For *Cx.* *quinquefasciatus*, the proportion of dead mosquitoes with LLINs decreased slightly but significantly (likelihood ratio test, p ≤ 0.05) with increasing holed surface area and ranged from 0 to 0.11. The regression model with just two explanatory variables (mosquito species and type of net) explained 97.9 % of the variation in the data (Table 5). With this model, the odds of *An.* *gambiae* KISUMU dying were 7330.5 times higher with LLINs than with untreated nets. For *An.* *gambiae* AKRON and *Cx.* *quinquefasciatus*, these values were 461.5 and 34.4, respectively. Addition of the logarithmically transformed holed surface area increased the proportion of explained variation by 0.1 percentage points, but this was not significant (likelihood ratio test, p > 0.05). Further addition of the hole size variable increased the explained variation to 98.9 % (likelihood ratio test, p ≤ 0.05).

For the proportion of mosquitoes in the veranda, the results for the crude outcomes are shown in Fig. 2 panels a, c, and e and in the Tables 2, 3 and 4 for all the tested species. The exit seeking proportion with untreated nets ranged from 0.40 to 0.69 for *An.* *gambiae* KISUMU, from 0.47 to 0.64 for *An.* *gambiae* AKRON and from 0.09 to 0.48 for *Cx.* *quinquefasciatus*. With LLINs, the exit seeking proportion ranged from 0.20 to 0.78 for *An.* *gambiae* KISUMU, from 0.62 to 0.79 for *An.* *gambiae* AKRON and from 0.38 to 0.78 for *Cx.* *quinquefasciatus*. The regression model with mosquito species and type of net explained 65.2 % of the variation in the data (Table 5). Addition of the logarithmically transformed holed surface area increased (likelihood ratio test, p ≤ 0.05) this by 11 percentage points from 65.2 % to a total of 76.2 %. Further addition of the hole size variable increased (likelihood ratio test, p ≤ 0.05) this by 12.5 percentage points from 76.2 to 88.7 %.

For the proportion of mosquitoes collected under the net, the results for the crude outcomes are shown in Fig. 2 panels b, d, and f and in the Tables 2, 3 and 4 for all tested species. The proportions under the untreated nets ranged from 0.01 to 0.24 for *An.* *gambiae* KISUMU, from 0 to 0.23 for *An.* *gambiae* AKRON and from 0 to 0.71 for *Cx.* *quinquefasciatus*. The proportion under LLINs ranged from 0 to 0.45 for *An.* *gambiae* KISUMU, from 0 to 0.17 for *An.* *gambiae* AKRON and from 0 to 0.44 for *Cx.* *quinquefasciatus*. For all mosquito species and net types, the proportions of mosquitoes found under the nets were largest at intermediate holed surface area. The regression model with mosquito species and type of net explained 39.8 % of the variation in the data (Table 5). Addition of the logarithmically transformed holed surface area increased (likelihood ratio test, p ≤ 0.05) this by 34.1 percentage points from 39.8 % to a total of 73.9 %. Further addition of the hole size variable increased (likelihood ratio test, p ≤ 0.05) this by 20.6 % points from 73.9 to 94.5 %. The ratio of the proportion of fed mosquitoes found under the net and the proportion of fed mosquitoes found elsewhere in the hut was high at low holed surface area and declined with increasing holed surface towards unity (Additional file 4). The ratio of the proportion of dead mosquitoes found under the net and the proportion of dead mosquitoes found elsewhere did not vary with the holed surface area in a clear pattern (Additional file 4). Overall, the risk of dying for a mosquito found under an LLIN versus a mosquito found elsewhere in the hut was slightly higher for *An.* *gambiae* AKRON (Risk ratio 1.41; 95 % CI 1.14–1.69) and slightly lower for *Cx.* *quinquefasciatus* (Risk ratio 0.52; 95 % CI 0.27–0.97).

### Mosquito release-recapture assay in huts: measures of protective effects

**Fig. 3:**
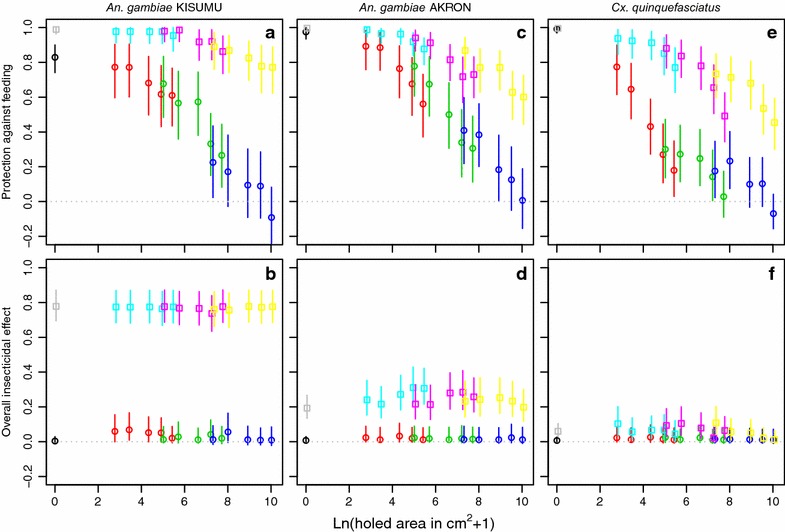
Direct bednet effects depending on the holed surface area on three mosquito species. The *first column of panels* **a**, **b** is for *An.* *gambiae* KISUMU strain, the *second column of panels* **c**, **d** is for *An.* *gambiae* collected in Akron, and the *third column of panels* **e**, **f** is for *Cx.* *quinquefasciatus* collected in Cotonou. The *first row of panels* **a**, **c**, **e** shows the personal protection against feeding (see "Appendix"), the *second row of panels* **b**, **d**, **f** shows the overall insecticidal effect of a bed net used by an individual. *Symbols* show median values with *grey squares* representing intact LLINs; *cyan squares* LLINs with holes of 3 cm^2^; *magenta squares* LLINs with holes of 30 cm^2^; *yellow squares* LLINs with holes of 300 cm^2^; *black circles* intact untreated nets; *red circles* untreated nets with holes of 3 cm^2^; *lime green circles* untreated nets with holes of 30 cm^2^; *dark blue circles* untreated nets with holes of 300 cm^2^. *Vertical lines* show 95 % confidence intervals.

**Fig. 4:**
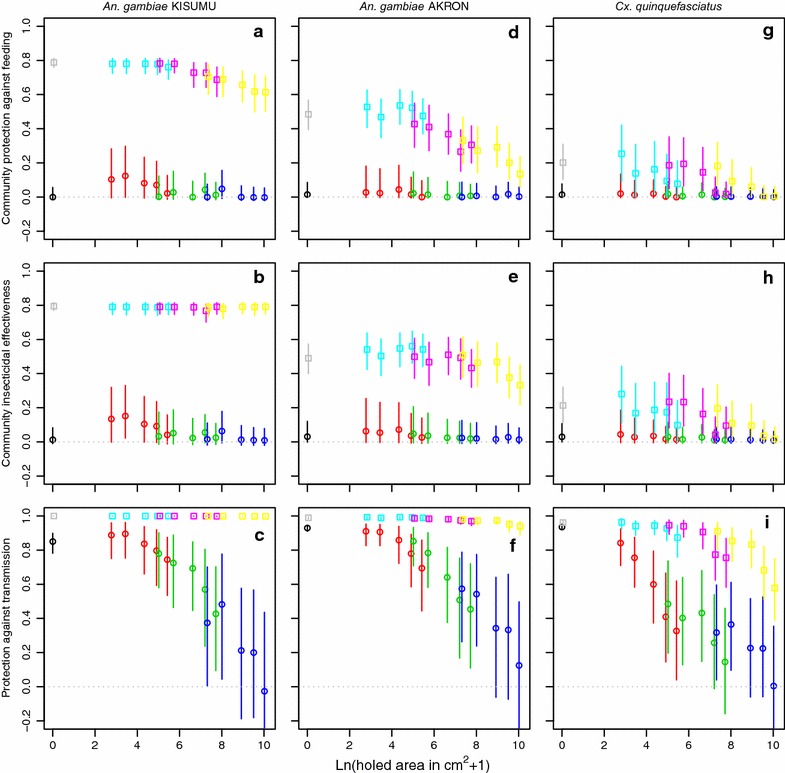
Community protective effects of 80 % bednet use coverage depending on the holed surface area on three mosquito species. The *first column of panels* **a**–**c** is for *An.* *gambiae* KISUMU strain, the *second column of panels* **d**–**f** is for *An.* *gambiae* collected in Akron, and the *third column of panels* **g**–**i** is for *Cx.* *quinquefasciatus* collected in Cotonou. The *first row of panels* **a**, **d**, **g** shows the community protection against feeding assuming that 80 % of the population uses a net (see "Appendix"), the *second row of panels* **b**, **e**, **h** shows the community insecticidal effectiveness, and the *third row of panels* **c**, **f**, **i** shows protection against transmission (note that this is theoretical for *Cx.* *quinquefasciatus*, as it is not a competent malaria vector). Legend further as in Fig. 3.

The results of the analysis of outcome measures are shown in Figs. 3 and 4. Consistent with the results for the crude proportion blood fed, personal protection against blood feeding (Fig. 3 panels a, c and e) decreased roughly linearly with the holed surface area on a logarithmic scale. Thus, protection decreased roughly exponentially with holed surface area on a linear scale. For *An.* *gambiae* KISUMU, the relationship was somewhat flat at a small holed surface area for LLINs. The (imaginary) curve describing the relationship for untreated nets was higher for *An.* *gambiae* AKRON than for *An.* *gambiae* KISUMU, suggesting that untreated nets consistently provided less protection against *An.* *gambiae* KISUMU. In contrast, LLINs appeared to protect relatively better against *An.* *gambiae* KISUMU than against *An.* *gambiae* AKRON as the holed surface area increased (Fig. 3 panels a, c). This was expected, as *An.* *gambiae* KISUMU is more susceptible to pyrethroids. Compared to *An.* *gambiae*, protection of untreated nets against feeding by *Cx.* *quinquefasciatus* quickly dropped as the holed surface area increased (Fig. 3 panel e), suggesting that *Cx.* *quinquefasciatus* was more capable of finding holes in nets than *An.* *gambiae*. With LLINs, protection against feeding dropped less quickly with increasing holed surface area (despite *Cx.* *quinquefasciatus* being highly pyrethroid resistant) than with untreated nets, but still faster than with *An.* *gambiae*.

Community protection against blood feeding (Fig. 4 panels a, d and g) at 80 % coverage was almost nil for untreated nets, irrespective of the holed surface area, as these nets diverted most mosquitoes to unprotected hosts. Community protection by LLINs against blood feeding was broadly consistent with the observations made for personal protection against feeding, with the exception that the level of protection was lower for resistant *An.* *gambiae* AKRON than for susceptible *An.* *gambiae* KISUMU, and much lower for highly resistant *Cx.* *quinquefasciatus*. Figure A5.1 in Additional file 5 shows how the community protection against blood feeding dropped when only 60 or 40 % of the population uses nets.

The overall insecticidal effect of a bed net used by an individual was almost nil for untreated nets (Fig. 3 panels b, d and f). The overall insecticidal effect of LLINs was high (nearly at 80 %) on susceptible *An.* *gambiae* KISUMU, irrespective of the holed surface area. It was much lower (at about 20 %) for *An.* *gambiae* AKRON, with a slight curve in the relationship, hinting that the overall insecticidal effect might be somewhat higher at intermediate holed surface area than at either end of the range (intact and very badly holed). For the highly resistant *Cx.* *quinquefasciatus*, the shape of the curve was similar, but the insecticidal effect was almost nil.

The predicted community level insecticidal effectiveness of a bed net programme at 80 % coverage (Fig. 4 panels b, e and h) showed similar curves as those of community protection against feeding, except that with LLINs for *An.* *gambiae* KISUMU the relationship was flat, i.e. predicted effectiveness did not decrease with increasing holed surface area. For *An.* *gambiae* AKRON, the relationship was less steep, i.e. predicted insecticidal effectiveness decreased less with increasing holed surface area than community protection against feeding did. Figure A5.2 in Additional file 5 shows how the community level insecticidal effect dropped when only 60 or 40 % of the population uses nets.

Curves for protection against transmission at 80 % population coverage with untreated nets (Fig. 4 panels c, f and i) were very similar to those for protection against blood feeding, dropping approximately linearly with increasing holed surface area on a logarithmic scale. However, for *An.* *gambiae* KISUMU, protection by LLINs against transmission was near 100 % irrespective of the holed surface area, and for *An.* *gambiae* AKRON, dropped only slightly with increasing holed surface area. Figure A5.3 in Additional file 5 shows how the protection against transmission dropped when only 60 or 40 % of the population uses nets. For instance, with 60 % coverage with new intact LLINs, 94 % reduction in transmission by *An.* *gambiae* AKRON was obtained, instead of 99 % reduction with 80 % population coverage of nets.

## Discussion

### Insecticide susceptibility

The WHO susceptibility test confirmed the susceptible status of *An.* *gambiae* KISUMU to 0.05 % deltamethrin and the resistant status of *An.* *gambiae* AKRON to this compound. Even though the WHO recommends a discriminating dose of (only) 0.025 % deltamethrin for *Cx.* *quinquefasciatus* [34], in this study, the susceptibility of the Cotonou *Cx.* *quinquefasciatus* population was tested against 0.05 % deltamethrin, resulting in only 20 % mortality, demonstrating the strong resistance of this mosquito population to this pyrethroid.

**Fig. 5:**
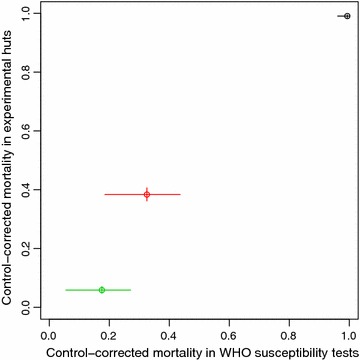
Mortality in WHO susceptibility tests and in experimental huts. The *black circle* represents *An.* *gambiae* KISUMU, the *red circle* represents *An.* *gambiae* AKRON and the *green circle* represents *Cx.* *quinquefasciatus*. *Vertical* and *horizontal lines* indicate 95 % confidence intervals.

The resistance measured in WHO susceptibility tests correlated well with resistance measured in the release-recapture trials with LLINs (Fig. 5). The somewhat lower than expected control-corrected mortality for *Cx.* *quinquefasciatus* in the experimental huts might be explained by the fact that LLINs were between two and three months old during the release-recapture trials with *Cx.* *quinquefasciatus*. Similarly, strong correlations between mortality in susceptibility tests and LLIN effectiveness measures were observed by Briët and colleagues [20]. Interestingly, the difference in feeding success with untreated nets as compared to LLINs was smaller for *An.* *gambiae* AKRON than for the more resistant *Cx.* *quinquefasciatus*. This may be related to the observation that LLINs elicited stronger exit-seeking behaviour (relative to untreated nets) in *Cx.* *quinquefasciatus* than in *An.* *gambiae* AKRON.

### Mosquito release–recapture assay in huts: effect of holes in nets

**Fig. 6:**
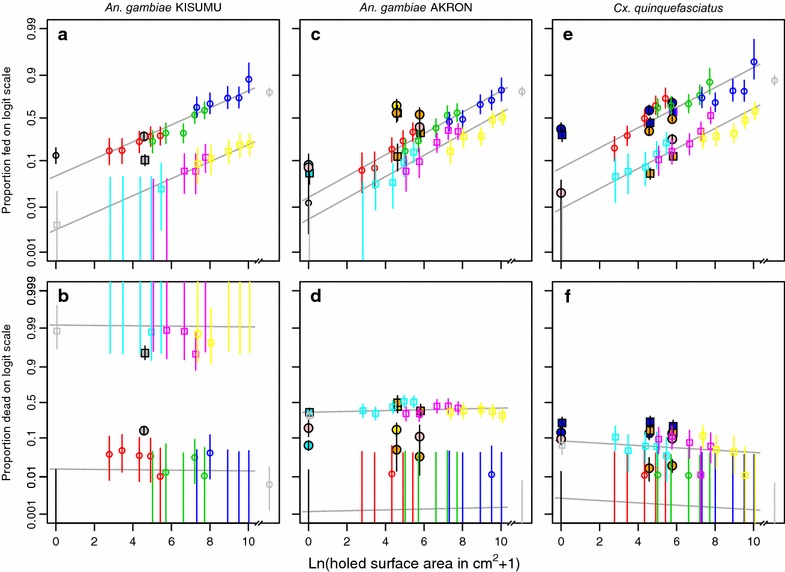
Proportions of mosquitoes fed, and dead depending on the holed surface area on three mosquito species in relation to data in literature. The *first column of panels* **a**, **b** is for *An.* *gambiae* KISUMU strain, the *second column of panels* **c**, **d** is for *An.* *gambiae* collected in Akron, and the *third column of panels* **e**, **f** is for *Cx.* *quinquefasciatus* collected in Cotonou. The *first row of panels* **a**, **c**, **e** shows the proportion fed, the *second row of panels* **b**, **d**, **f** shows the proportion dead. *Symbols* show mean values with *grey squares* representing intact LLINs; *cyan squares* LLINs with holes of 3 cm^2^; *magenta squares* LLINs with holes of 30 cm^2^; *yellow squares* LLINs with holes of 300 cm^2^; *black circles* intact untreated nets; *red circles* untreated nets with holes of 3 cm^2^; *lime green circles* untreated nets with holes of 30 cm^2^; *dark blue circles* untreated nets with holes of 300 cm^2^. *Grey circles* represent the results from huts without nets. *Vertical lines* show 95 % confidence intervals, and *grey lines* show the fitted relationships from logistic regressions with net type, mosquito species and holed surface area as explanatory variables. *Larger symbols* with *black outlines* represent data from literature with *fill colour grey* from Tungu and colleagues [27], *fill colour yellow* from N’Guessan and colleagues [29], *fill colour cyan* from Koudou and colleagues [60], *fill colour orange* from Ngufor and colleagues [36], *fill colour pink* from Asidi and colleagues [37], and *fill colour dark blue* from Irish and colleagues [35].

Generally, experimental huts studies with different sizes or numbers of holes, and studies with intact and holed nets show that, with increased holed surface area, there is increased blood feeding [35]. Most studies included up to two categories of holed surface area, e.g. [36, 37]. One experimental hut study by Irish and colleagues [14] examined the effect of holed surface area in three categories on *Cx.* *quinquefasciatus*. The present study, with nets in 16 categories of physical condition, as well as a no net control, shows that the logit transformed probability of feeding for both *An.* *gambiae* and *Cx.* *quinquefasciatus* increases approximately linearly with the logarithmically transformed holed surface area. A direct comparison with data from a few selected experimental hut studies with voluntarily entering mosquitoes is provided in Fig. 6. Data from Asidi and colleagues [37] for both *An.* *gambiae* and *Cx.* *quinquefasciatus*, and from Ngufor and colleagues [36] for *Cx.* *quinquefasciatus* and *An.* *gambiae* (for treated nets) suggest relationships with similar slopes as found in this study, albeit with sometimes different offset. However, Ngufor and colleagues [36] did not observe a difference between two categories of holed nets in their effect on feeding of *An.* *gambiae*, and the slopes found by Irish and colleagues [14] are weaker due to a higher proportion of fed mosquitoes with intact untreated and treated nets. The personal protection against feeding decreased almost linearly with the logarithmically transformed holed surface area. This implies that personal protection against feeding decreased roughly exponentially with the holed surface area. Because personal protection against feeding decreased gradually with hole area, there is no clear holed surface area cut‐off that could distinguish between serviceable and non‐serviceable LLINs, such as that proposed by WHOPES [16]. In fact, the regression analysis suggested that completely ragged LLINs containing the target insecticide concentration could still significantly prevent mosquitoes from feeding (if the linear relationship found for LLINs with a holed surface area in the range of 0–15.5 % could be extrapolated to 100 %), despite the absence of a physical barrier. Although not a complete lack of physical barrier, Curtis and colleagues showed that permethrin-impregnated bed curtains made from polypropylene sacks without a top panel (in this study, this would correspond to about 23.5 % holed surface area) significantly reduced feeding by *An.* *gambiae* and *Anopheles* *funestus* [38]. Some proximity of the impregnated material to the sleeper would still be required, as the same authors [39] did not find a significant reduction in blood feeding with deltamethrin-impregnated cotton wall curtains. The physical state of nets had virtually no influence on their insecticidal effect, since badly holed nets and intact nets killed similar proportions of released mosquitoes. This is consistent with Ngufor and colleagues [36], who observed no relationship between mortality of *An.* *gambiae* and of *Cx.* *quinquefasciatus* and holed surface area in nets in Akron, with Irish and colleagues [14] who, working in Ladji (Cotonou), also observed that the mortality rate for *Cx.* *quinquefasciatus* was not significantly different between nets with different numbers of holes, and with Asidi and colleagues [37] who, working in Yaokoffikro in Côte d’Ivoire, observed no difference in mortality of *An.* *gambiae* and *Cx.* *quinquefasciatus* with intact untreated and holed untreated nets.

The effect of nets on malaria transmission is a combination of both their effect on protection against feeding and their insecticidal effect. Modelling indicates that as long as the insecticide concentration is high (as in the up to three months old PermaNet 2.0 nets used in this experiment), badly holed LLINs with 22,500 cm^2^ (15.5 %) holed surface area could still reduce transmission (compared to a situation without nets) by the highly deltamethrin-resistant *An.* *gambiae* AKRON by 94 % (95 % CI 89–97 %) if such nets are used by 80 % of the population. With 60 % net use, transmission would be reduced by 94 % (95 % CI 92–96 %) with intact and by 85 % (95 % CI 78–90 %) with badly holed LLINs. With 40 % net use, transmission would be reduced by 80 % (95 % CI 75–85 %) with intact and by 69 % (95 % CI 60–76 %) with badly holed LLINs compared to a situation without nets. Apparently, the low mortality of pyrethroid resistant mosquitoes may be still sufficient to prevent most of them from surviving the extrinsic incubation period. If *An.* *gambiae* attains a resistance profile similar to that of *Cx.* *quinquefasciatus* in Cotonou, badly holed nets (still containing the target insecticide concentration) would only reduce transmission by 58 % (95 % CI 39–75 %) with 80 % net use in the population. The predicted effectiveness of badly holed LLINs with 22,500 cm^2^ appears to be at odds with a proposed thresholds for classifying LLINs with more than 790 cm^2^ of holed surface area with round holes in a category where “protective efficacy for the user is in serious doubt” [18]. Clearly, protective efficacy depends not only on the holed surface area, but also on the hole size, amount of insecticide left in the net, and insecticide susceptibility of the vector.

There was no clear relationship between the holed surface area in nets and the proportion of mosquitoes found in the veranda, with holed surface area explaining only 11 % out of a total of 76.2 % of variation explained by a model incorporating species, net type and holed surface area. Fewer *Cx.* *quinquefasciatus* mosquitoes were found in the veranda with untreated nets at intermediate holed surface area, and with LLINs at high holed surface area, but there was no difference between arms of no net and intact untreated nets, suggesting that a part of this effect could be due to mosquitoes being trapped under the net.

The relationship between the holed surface area in nets and the proportion of mosquitoes found under the net was non-monotonic, with a maximum at intermediate holed surface area. Holed surface area was an important determinant of the proportion of mosquitoes under the net, explaining 34.1 % out of a total of 73.9 % of variation explained by a model incorporating species, net type and holed surface area. The fact that addition of the hole size variable augmented this with 20.6–94.5 % can be in large part attributed to the hole size variable allowing piece-wise linear fitting of the non-monotonic relationship, due to only partially overlapping holed surface areas for the arms with different hole sizes, and not to there being a large difference in mosquitoes under the net with different hole sizes at a similar total holed surface area. The proportion of mosquitoes found under the net is probably a function of the rate of unfed mosquitoes entering the net, the duration of resting inside the net after feeding, the rate of (mostly) fed mosquitoes exiting the net, and the duration of the experiment. At the higher end of the holed surface area range, most mosquitoes that wanted to feed had done so and had left the net by the end of the experiment, resulting in the declining number of mosquitoes found under the net with holed surface area at the higher end of the range. This explanation is supported by the observation that the ratio of the proportion of fed mosquitoes found under the net and the proportion of fed mosquitoes found elsewhere was high at low holed surface area and declined with increasing holed surface area (Additional file 4). Holed surface area did not appear to have an effect on the ratio of the proportion of dead mosquitoes found under the net and the proportion of dead mosquitoes found elsewhere. Lines and colleagues [40] observed that mosquitoes found under untreated nets are less likely to be dead than mosquitoes found elsewhere, and that the reverse is true with treated nets. In the present study, this could only be assessed for LLINs, as mortality was minimal for untreated nets. If found under LLINs, the more resistant *Cx.* *quinquefasciatus*, was less likely to be dead than if found elsewhere, whereas the less resistant *An.* *gambiae* AKRON was more likely to be dead if found under the net than if found elsewhere.

The effect of hole size on feeding was small but significant, explaining 5.1 % out of 97.9 % of total explained variation in the regressions. The effect of hole size was strongest for *An.* *gambiae* AKRON and *Cx.* *quinquefasciatus*, with the proportion of fed mosquitoes of these species being (slightly) larger if the holes were smaller in size (but more numerous) than if the holes were larger in size (but smaller in number), at the same total holed surface area. Whether the small difference in effect of hole size at the same total holed surface area is sufficient to merit reporting numbers of holes in separate categories is debatable. However, it is clear that evaluations of physical status of LLIN populations in the field should try to capture the approximate total holed surface area, whether made up from a few large holes, or many small holes [41]. It may be that holes in the top panel are more important than holes in the side panels, but the design of this study did not allow evaluating this. In recent behavioural studies, it was found that much of the activity of *An.* *gambiae* occurs at the roof [42, 43]. This may lead to recommendations of recording the location of holes when assessing net damage. Further experimental hut studies are needed to determine how location of holes in nets, and how lower insecticide concentrations in older nets affect feeding, mortality and malaria transmission by resistant mosquitoes.

### Mosquito release-recapture assay in huts: biases with released insectary-raised mosquitoes and with voluntarily entering wild mosquitoes

In this study, mosquitoes that had pupated and hatched in insectaries were released in batches of roughly the same age inside closed experimental huts. In the literature, most studies on the effect of nets on mosquito mortality and feeding on humans use experimental huts in the vicinity of mosquito breeding sites where mosquitoes enter huts voluntarily. There could be important differences between voluntarily entering mosquitoes and insectary-reared released mosquitoes. Voluntarily entering mosquitoes are likely to be more heterogeneous in physical condition due to difference in physical age, parity, and previous feeding success on blood or nectar sources. Some voluntarily entering mosquitoes may also be engorged or gravid prior to entry and merely be looking for a resting site [44]. In such circumstances, identifying mosquitoes that fed elsewhere is not straight forward [45, 46], and gravid mosquitoes are not always excluded from the analysis of experimental hut data. In contrast, 4–5 day-old never-blood fed females are more likely to be uniformly in a state of host seeking. In this study, in the arm without a net, feeding rates were 78.9, 79.6 and 87.3 % for *An.* *gambiae* KISUMU, *An.* *gambiae* AKRON and *Cx.* *quinquefasciatus*, respectively. Thus, still 13–21 % of mosquitoes presumably in a similar condition did not feed despite the presence of an unprotected host. Few recent experimental hut studies include a no-net arm for comparison of effects, probably because human volunteers in those arms might be more exposed to potentially infectious mosquitoes in such an experiment arm than if not participating in the study. Older studies generally report feeding rates in arms without nets in the range of 75–95 % [38, 40, 45, 47], and in some experiments, rates of 96–100 % [10] were found.

In this study, mortality in arms without nets was very low at 0.6, 0 and 0 % for *An.* *gambiae* KISUMU, *An.* *gambiae* AKRON and *Cx.* *quinquefasciatus*, respectively. In studies with voluntarily entering wild mosquitoes, mortality rates are much higher in the range of 9–51 % [38, 40, 45, 47], although in one location (Magugu), Smith and Webley [47] recorded only 2 % mortality in *An.* *gambiae* s.l. It is possible that mosquitoes that are in need of a blood meal but fail to obtain one are more likely to die than mosquitoes that feed. In this study, within study arms, no difference in the probability of dying was found between those mosquitoes that were unfed and those that had managed to obtain a blood meal (Additional file 6). However, data from voluntarily entering *An.* *gambiae* in Akron [29] and *Cx.* *quinquefasciatus* in Ladji, near Cotonou [14] showed a higher risk of dying for unfed mosquitoes than for those mosquitoes that managed to feed. It is not known whether unfed mosquitoes were more likely to die because they did not feed, or if the weaker among the mosquitoes failed to feed and died because they were weaker. In this study, with no apparent effect of feeding status on survival and very low mortality in the absence of insecticide, such heterogeneity or an effect of feeding on survival was likely to be of lesser importance than in studies with voluntarily entering mosquitoes. Another difference between the two methods is in the length of exposure to the treatments. In all-night experiments with voluntarily entering mosquitoes, some mosquitoes may enter late in the night, and have little exposure to the intervention. In contrast, mosquitoes simultaneously released inside huts early in the evening are all exposed to the treatment for the same period.

Despite efforts to trap voluntarily entering mosquitoes inside the experimental huts with the help of baffles, mosquitoes do escape from experimental huts, as evidenced in tests with mosquitoes released inside experimental huts [10]. Such escape could heavily bias tests of effectiveness of indoor interventions. Unfed (alive) mosquitoes are most likely to escape, as engorged mosquitoes are less active [48] and less likely to leave the hut [10, 44]. Tests with interventions that prevent feeding may underestimate the total number of entered mosquitoes, and thus underestimate the protective effect against feeding, and overestimate the insecticidal effect. Further, escape from huts may be more likely with repellent non-lethal interventions than with lethal non-repellent interventions, and experimental assays are thus likely to underestimate the efficacy of repellent non-lethal interventions, especially if deterrence from hut entry (which is likely to be larger for repellent interventions) cannot be taken into account, for instance when treatment rotation over huts cannot be done. In experiments with released mosquitoes with closed entry slits, escape is likely much lower than in experiments with voluntarily entering mosquitoes, although escape through cracks and doors and other losses (due to predation) are expected to be similar to those occurring in experiments with voluntarily entering mosquitoes. Such biases might be responsible for some of the differences in results of this study and those of experiments with mosquitoes entering huts voluntarily.

There are several factors that influence a mosquito’s ability to feed on a human protected by a net, including the presence of holes in the net, the mosquito’s resistance to the insecticide on the net and the proximity of the human to the net (determined by the size of the net, the number of people inside the net, the sleeping location of the human, etc.), which could allow feeding through the net. The ‘intrinsic’ behaviour of the mosquito may also play an important role. In this study, the relatively high proportion of blood fed females of the laboratory strain *An.* *gambiae* KISUMU with intact untreated nets compared with virtually no feeding of females from the *An.* *gambiae* AKRON population (and from the *Cx.* *quinquefasciatus* population) with intact untreated nets is noteworthy. As the laboratory strain *An.* *gambiae* KISUMU has been fed for many years on defenceless rabbits placed directly on top of their mesh cages, it is possible that this strain has lost some of its restraint from directly approaching hosts, and has become skilled in feeding through mesh material. The high proportion (36.2 %) of fed mosquitoes in the presence of untreated intact nets in experiments with *Cx.* *quinquefasciatus* voluntarily entering experimental huts [14] contrasts sharply with the complete lack of feeding in this study by released *Cx.* *quinquefasciatus* from the same area. As intrinsic behaviour differences are unlikely, these differences could be explained by a proportion of entering mosquitoes being already engorged, or perhaps due to smaller nets being used in those experiments, allowing for more skin contact with the nets.

### Implications for net distribution rates

In a naturally decaying LLIN population, nets will both gain holes and lose insecticide as they age, with large differences in the rate at which this happens depending on the individual users and household conditions. Moreover, net care and repair behaviours are likely to impact on net longevity and durability by prolonging the protective lifespan of LLINs [17, 49, 50]. Some nets might have gained holes quickly while still containing a high concentration of insecticide and other nets might be virtually intact but with little insecticide left due to washing and handling [51]. Both types of nets described above may provide substantial protection against malaria transmission as compared to heavily torn nets containing a low residual concentration of insecticide, or no net. A “failed net” is a net that is no longer being used for its intended purpose. Net use has been strongly correlated with net condition [52]. Even if a holed net may continue to kill mosquitoes and thus could contribute to malaria control, the physical appearance of the net and the presence of (nuisance) mosquitoes in the net may lead people to retire the nets from their intended use [53, 54]. Loll and colleagues [54] suggest developing user guidelines for determining the combined state of physical and chemical deterioration that a net should be retired at.

Providing universal coverage of LLINs to populations at risk has become a priority for NMCPs in recent years [55]. For an NMCP that relies on LLINs for malaria transmission prevention, not personal protection, but community level protection against transmission is important. From the perspective of NMCPs, the distribution rate of new nets should be such that the general effectiveness of the net population, as a whole, is satisfactory. Combining data on effects on personal protection and insecticidal efficacy on use rates, attrition rates, insecticide decay rates, the rate of hole formation and insecticide resistance data in a mathematical model could provide NMCPs with better guidance on optimal LLIN distribution and replacement. Further, such models could be used to determine whether the decisions of net owners to retire their nets are appropriate, or whether influence should be exerted to, for example, extend the life of nets as long as possible to bridge coverage gaps.

## Conclusions

Although holes in LLINs reduce the personal protection against feeding mosquitoes, the insecticidal effect of LLINs is independent of the holed surface area but strongly depends on insecticide resistance of the mosquito population. This study suggests that badly torn nets (22,500 cm^2^ holed surface area) that contain insecticide at concentrations of up to three months old LLINs still reduce malaria transmission, if used. LLIN distribution policies targeting transmission reduction should not be based on an arbitrary threshold for the holed surface area in the LLIN, but instead be based on a ‘transmission-reducing effectiveness index’ of the LLIN population, which is a function of LLIN use rates, the physical state of the LLIN population, the chemical state of the LLIN population and behavioural and insecticide resistance in the local vector populations.

## Appendix: measures of protective effects of (indoor) vector control interventions

### Protection against feeding

In experimental hut trials, the personal protection against feeding $\varPhi$ is calculated as $\varPhi = 1 - \frac{{F_{t} }}{{F_{0} }}$ [56] with *F*_*t*_ the number of fed females in the treatment arm, and *F*_0_ the number of fed females in the untreated arm. If the treatment involves an insecticide, like with an LLIN, *F*_*t*_ is expected to be smaller than *F*_0_ because of repellence from feeding, pre-prandial killing and deterrence from hut entry. Deterrence from hut entry is presumed to be caused by odours of the insecticide emanating from the hut, and is presumably unaffected by the state of physical damage of nets. Because in this experiment, mosquitoes were not freely entering the huts, but released inside closed huts, the estimate of $\varPhi$ has to be corrected for deterrence from hut entry, $\delta$, which can be estimated from other experiments. Deterrence, the proportionate reduction in the entry rate, is defined as $\delta = 1 - \frac{{E_{t} }}{{E_{0} }}$ with $E_{t}$ the number of females that entered the hut in the treatment arm, and $E_{0}$ the number of females that entered the hut in the untreated arm. With the feeding success $\varphi_{0} = {{F_{0} } \mathord{\left/ {\vphantom {{F_{0} } {E_{0} }}} \right. \kern-0pt} {E_{0} }}$ and $\varphi_{t} = {{F_{t} } \mathord{\left/ {\vphantom {{F_{t} } {E_{t} }}} \right. \kern-0pt} {E_{t} }}$ with $F_{t}$ the number of fed females in the treatment arm, $F_{0}$ the number of fed females in the untreated arm,

1 $$ \varPhi = 1 - \frac{{F_{t} }}{{F_{0} }} = 1 - \frac{{E_{t} \varphi_{t} }}{{E_{0} \varphi_{0} }} = 1 - \frac{{\left( {1 - \delta } \right)\varphi_{t} }}{{\varphi_{0} }}. $$

Feeding that may occur after mosquitoes are diverted to other hosts is ignored by this efficacy measure.

### Community protection against feeding

In programmatic applications, the community level protection against feeding depends on, in addition to the personal protection against feeding, the coverage, $c$, since mosquitoes diverted from a treated hut may, instead of finding a blood meal in an untreated hut, encounter another treated hut with probability $c$, so that the feeding success decreases with coverage.

As explained previously [57], the community level protection against feeding $\varPhi_{c}$ (there called “population level protection against feeding”) in an intervention programme is a function of the average feeding success when a proportion of the human population is protected, and the feeding success when no-one is protected:

$$ \varPhi_{c} = \frac{{\varphi_{0}^{{ \star }} - \bar{\varphi }_{c}^{{ \star }} }}{{\varphi_{0}^{{ \star }} }} $$

where $\bar{\varphi }_{c}^{{ \star }} = \frac{{c\left( {1 - \delta } \right)\left( {1 - \rho_{t} } \right)\varphi_{t}^{{ \star }} + \left( {1 - c} \right)\left( {1 - \rho_{0} } \right)\varphi_{0}^{{ \star }} }}{{c\left( {1 - \delta } \right)\left( {1 - \rho_{t} } \right) + \left( {1 - c} \right)\left( {1 - \rho_{0} } \right)}}$ is the average feeding success when a proportion of the human population is protected, with $\rho_{0} = {{U_{0} } \mathord{\left/ {\vphantom {{U_{0} } {E_{0} }}} \right. \kern-0pt} {E_{0} }}$ the proportion of females repelled after entry in the control arm, $\rho_{t} = {{U_{t} } \mathord{\left/ {\vphantom {{U_{t} } {E_{t} }}} \right. \kern-0pt} {E_{t} }}$ the proportion of females repelled after entry in the treated arm, $U_{0}$ the number of unfed alive mosquitoes in the control arm, $U_{t}$ the number of unfed alive mosquitoes in the treated arm, $\varphi_{0}^{{ \star }} = {{\varphi_{0} } \mathord{\left/ {\vphantom {{\varphi_{0} } {\left( {1 - \rho_{0} } \right)}}} \right. \kern-0pt} {\left( {1 - \rho_{0} } \right)}}$ is the feeding success in the control arm out of those that were not repelled, $\delta$ and $E_{t}$ are as defined under ‘Protection against feeding’ and $\varphi_{t}^{{ \star }} = {{\varphi_{t} } \mathord{\left/ {\vphantom {{\varphi_{t} } {\left( {1 - \rho_{t} } \right)}}} \right. \kern-0pt} {\left( {1 - \rho_{t} } \right)}}$ is the feeding success with the intervention out of the proportion that was not repelled. Note that repellence is defined here as the proportion of both unfed and live mosquitoes out of those that entered the hut and is not necessarily equal to the proportion of mosquitoes found in exit traps (including veranda traps). All other mosquitoes collected (dead or both alive and fed) are presumed to have attacked the occupant of the hut. Also note that $\varPhi_{c}$ assumes that mortality during host searching is negligible and can be biased if a proportion of the mosquitoes that enter huts is exophagic. Also, note that possible sub‐lethal behavioural effects of interventions that interfere with further host location and feeding are not considered.

### Overall insecticidal effect of an individual treated hut

The overall insecticidal effect of a treated hut, corrected for mortality in the untreated hut (mosquitoes that would die anyway do not contribute to the insecticidal effect) is: $\varPsi = \frac{{\bar{\mu }^{{ \star }} - \mu_{0}^{{ \star }} }}{{1 - \mu_{0}^{{ \star }} }} = \left( {\frac{{\mu_{t}^{{ \star }} - \mu_{0}^{{ \star }} }}{{1 - \mu_{0}^{{ \star }} }}} \right)\left( {1 - \delta } \right)\left( {1 - \rho_{t} } \right)$, with $\mu_{t}^{{ \star }} = {{D_{t} } \mathord{\left/ {\vphantom {{D_{t} } {\left( {E_{t} - U_{t} } \right)}}} \right. \kern-0pt} {\left( {E_{t} - U_{t} } \right)}}$ the proportion of dead mosquitoes out of those that were not repelled after entry in treated arms, $\mu_{0}^{{ \star }} = {{D_{0} } \mathord{\left/ {\vphantom {{D_{0} } {\left( {E_{0} - U_{0} } \right)}}} \right. \kern-0pt} {\left( {E_{0} - U_{0} } \right)}}$ the proportion of dead mosquitoes out of those that were not repelled after entry in untreated arms, and $D_{t}$ and $D_{0}$ represent the number of dead mosquitoes in treated and untreated arms, respectively [57].

### Community insecticidal effectiveness

Like with the community level protection against feeding, in programmatic applications, the community level insecticidal effectiveness depends on the overall insecticidal effect and the coverage, since mosquitoes diverted from a treated hut may encounter another treated hut [57]. Assuming that no shifts to outdoor biting and resting occur, that there is no additional mortality while host-seeking and that treated and untreated huts are perfectly mixed, the mean mosquito mortality is $\bar{\mu }_{c}^{{ \star }} = \frac{{c\left( {1 - \delta } \right)\left( {1 - \rho_{t} } \right)\mu_{t}^{{ \star }} + \left( {1 - c} \right)\left( {1 - \rho_{0} } \right)\mu_{0}^{{ \star }} }}{{c\left( {1 - \delta } \right)\left( {1 - \rho_{t} } \right) + \left( {1 - c} \right)\left( {1 - \rho_{0} } \right)}}$ and the community level insecticidal effectiveness is $\varPsi_{c} = \frac{{\bar{\mu }_{c}^{{ \star }} - \mu_{0}^{{ \star }} }}{{1 - \mu_{0}^{{ \star }} }}$.

### Protection against transmission

The measures $\varPsi_{c}$ and $\varPhi_{c}$ are attractive because they make relatively few assumptions. However, the effectiveness of an intervention programme against parasite transmission depends on both, and a combined measure is more attractive. More sophisticated models, such as presented by Chitnis and colleagues [19], or by others [58, 59] can use both effects to predict protection against transmission, but need additional data or must make assumptions on parameter values. Protection against transmission of an intervention, with coverage $c$, is defined as $\varXi_{c} = 1 - \frac{{\chi_{c} }}{{\chi_{0} }},$ with $\chi_{c}$ the predicted entomological inoculation rate (EIR) with the intervention, and $\chi_{0}$ the EIR in the same epidemiological setting, without intervention.

## Additional files

Additional file 1: Hole position for each hole number per panel. Additional file 2: Data from experiments. Additional file 3: Regression model selection results. Additional file 4: Risk ratios of mosquitoes being fed or dead depending on location. Additional file 5: Figures for population effects against feeding, community insecticidal effectiveness and population effect on transmission depending on percent net use. Additional file 6: Risk ratios of mosquitoes being dead depending on being fed or unfed.
