# Supplementary material for: Do holes in long-lasting insecticidal nets compromise their efficacy against pyrethroid resistant Anopheles gambiae and Culex quinquefasciatus? Results from a release–recapture study in experimental huts
Source: Malar J. 2015 Aug 28;14:332. doi: 10.1186/s12936-015-0836-7 (PMC4551388; doi:10.1186/s12936-015-0836-7)
Supplement: Additional file 1: — Hole position for each hole number per panel. [file 12936_2015_836_MOESM1_ESM.pdf]

Additional file 1: Hole position for each hole number per panel

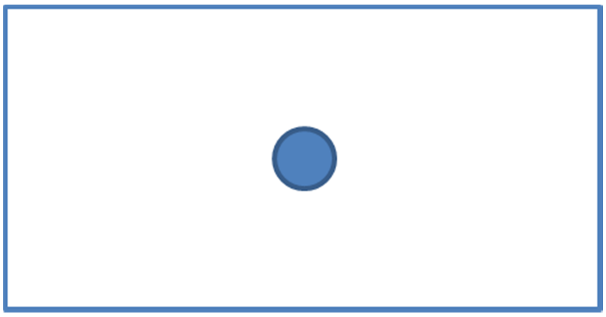

Figure 1.1 One hole per panel

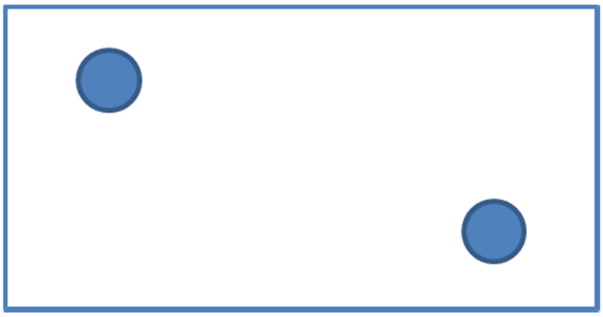

Figure 1.2 Two holes per panel

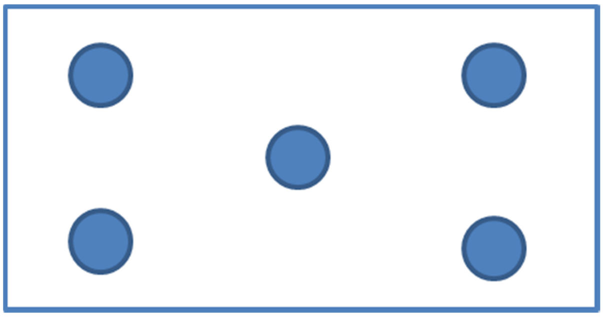

Figure 1.3 Five holes per panel

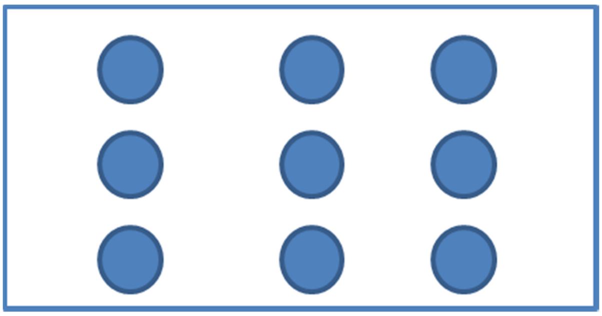

Figure 1.4 Nine holes per panel

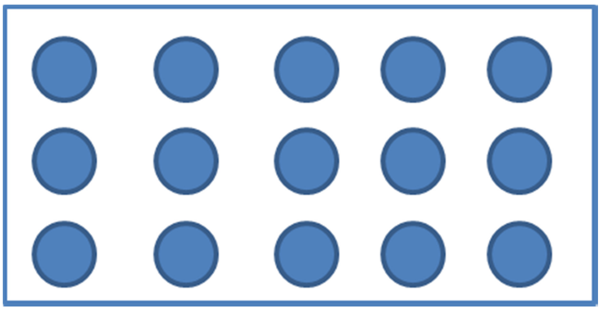

Figure 1.5 Fifteen holes per panel
