# Supplementary material for: Do holes in long-lasting insecticidal nets compromise their efficacy against pyrethroid resistant Anopheles gambiae and Culex quinquefasciatus? Results from a release–recapture study in experimental huts
Source: Malar J. 2015 Aug 28;14:332. doi: 10.1186/s12936-015-0836-7 (PMC4551388; doi:10.1186/s12936-015-0836-7)
Supplement: Additional file 3: — Regression model selection results. [file 12936_2015_836_MOESM3_ESM.pdf]

Additional file 3 Regression model selection results

Regression output from the function summary.glm in the statistical software package R

Legend

Fbin: matrix of 96 rows by 2 columns with the number of fed mosquitoes in the first column and the number of unfed mosquitoes in the second column.

Dbin: matrix of 96 rows by 2 columns with the number of dead mosquitoes in the first column and the number of live mosquitoes in the second column.

Verandabin: matrix of 96 rows by 2 columns with the number of mosquitoes collected in the veranda in the first column and the number of mosquitoes collected elsewhere in the second column.

Netbin: matrix of 96 rows by 2 columns with the number of mosquitoes collected under the net in the first column and the number of mosquitoes collected elsewhere in the second column.

Species: mosquito species in the categories 0 (*An. gambiae* KISUMU strain), 1 (*An. gambiae* collected in Akron), and 2 (*Cx. quinquefasciatus* collected in Cotonou).

Type: net type in the categories 0 (untreated net) and 1 (new PermaNet 2.0 LLIN).

Size: hole size in square cm in the categories 0, 3, 30 and 300.

logTHSp1: log of total holed surface area in cm^2 plus 1.

```
glm(formula = Fbin ~ Population + Type + Species:Type, family = binomial)
Deviance Residuals:
     Min        1Q    Median        3Q       Max
-20.8075   -3.0898    0.1389    3.7178    8.9589
Coefficients:
                  Estimate Std. Error z value Pr(>|z|)
(Intercept)       -0.43758    0.05364  -8.158  3.4e-16 ***
Species1       -0.27392    0.07710  -3.553 0.000381 ***
Species2        0.66044    0.07340   8.998  < 2e-16 ***
Type1             -2.36794    0.12450 -19.019  < 2e-16 ***
Species1:Type1  1.47324    0.14985   9.832  < 2e-16 ***
Species2:Type1  0.84869    0.14728   5.762  8.3e-09 ***
---
Signif. codes:  0 '***' 0.001 '**' 0.01 '*' 0.05 '.' 0.1 ' ' 1
(Dispersion parameter for binomial family taken to be 1)
    Null deviance: 3720.8  on 95  degrees of freedom
```

```
Residual deviance: 2435.3  on 90  degrees of freedom
AIC: 2823.8
Number of Fisher Scoring iterations: 5
```

```
glm(formula = Fbin ~ Species + logTHSp1 + Type + Species:Type +
      Species:logTHSp1, family = binomial)
Deviance Residuals:
    Min       1Q   Median       3Q      Max
-6.5595  -1.1565  -0.2264   0.8043   4.4321
Coefficients:
                     Estimate Std. Error z value Pr(>|z|)
(Intercept)          -2.90376    0.16397 -17.709  < 2e-16 ***
Species1          -1.08711    0.23880  -4.552  5.3e-06 ***
Species2           0.42576    0.20945   2.033  0.04208 *
logTHSp1              0.42187    0.02432  17.343  < 2e-16 ***
Type1                -2.76852    0.13607 -20.346  < 2e-16 ***
Species1:Type1     1.64802    0.16687   9.876  < 2e-16 ***
Species2:Type1     0.66696    0.16816   3.966  7.3e-05 ***
Species1:logTHSp1  0.10607    0.03397   3.122  0.00179 **
Species2:logTHSp1  0.07642    0.03200   2.388  0.01694 *
---
Signif. codes:  0 ‘***’ 0.001 ‘**’ 0.01 ‘*’ 0.05 ‘.’ 0.1 ‘ ’ 1
(Dispersion parameter for binomial family taken to be 1)
    Null deviance: 3720.78  on 95  degrees of freedom
Residual deviance:  266.99  on 87  degrees of freedom
AIC: 661.44
Number of Fisher Scoring iterations: 5
```

```
glm(formula = Fbin ~ Species + logTHSp1 + Type + Size + Species:Type +
      Species:Size + logTHSp1:Size + Species:logTHSp1 + logTHSp1:Type +
      Species:logTHSp1:Size, family = binomial)
Deviance Residuals:
     Min        1Q    Median        3Q       Max
-2.04485  -0.51391   0.01078   0.47215   2.21306
Coefficients: (3 not defined because of singularities)
                               Estimate Std. Error z value Pr(>|z|)
(Intercept)                  -1.934e+00  1.921e-01 -10.068  < 2e-16 ***
Species1                  -2.648e+00  6.120e-01  -4.327 1.51e-05 ***
Species2                  -1.912e+01  1.333e+03  -0.014 0.988559
logTHSp1                      3.674e-01  9.643e-02   3.809 0.000139 ***
```

```
Type1                        -3.413e+00  2.943e-01 -11.596  < 2e-16 ***
Size3                        -9.076e-01  5.979e-01  -1.518 0.129027
Size30                       -2.898e+00  7.364e-01  -3.936 8.29e-05 ***
Size300                      -2.495e-01  8.637e-01  -0.289 0.772700
Species1:Type1             1.790e+00  1.757e-01  10.187  < 2e-16 ***
Species2:Type1             8.881e-01  1.773e-01   5.009 5.48e-07 ***
Species1:Size3            -4.171e-02  1.065e+00  -0.039 0.968760
Species2:Size3             1.806e+01  1.333e+03   0.014 0.989192
Species1:Size30            2.054e+00  1.120e+00   1.833 0.066767 .
Species2:Size30            2.129e+01  1.333e+03   0.016 0.987259
Species1:Size300           3.541e-01  1.232e+00   0.287 0.773828
Species2:Size300           1.868e+01  1.333e+03   0.014 0.988823
logTHSp1:Size3               -5.955e-03  1.576e-01  -0.038 0.969867
logTHSp1:Size30               3.143e-01  1.414e-01   2.223 0.026215 *
logTHSp1:Size300                     NA         NA      NA       NA
Species1:logTHSp1          1.911e-01  1.215e-01   1.573 0.115785
Species2:logTHSp1          9.438e-02  1.230e-01   0.767 0.442990
logTHSp1:Type1                6.690e-02  3.309e-02   2.021 0.043241 *
Species1:logTHSp1:Size3    3.479e-01  2.222e-01   1.565 0.117486
Species2:logTHSp1:Size3    4.385e-01  2.018e-01   2.173 0.029761 *
Species1:logTHSp1:Size30  -1.057e-01  1.830e-01  -0.577 0.563797
Species2:logTHSp1:Size30  -2.103e-01  1.791e-01  -1.175 0.240136
Species1:logTHSp1:Size300         NA         NA      NA       NA
Species2:logTHSp1:Size300         NA         NA      NA       NA
---
Signif. codes:  0 '***' 0.001 '**' 0.01 '*' 0.05 '.' 0.1 ' ' 1
(Dispersion parameter for binomial family taken to be 1)
    Null deviance: 3720.776  on 95  degrees of freedom
Residual deviance:   76.934  on 71  degrees of freedom
AIC: 503.38
Number of Fisher Scoring iterations: 16


glm(formula = Dbin ~ Species + Type + Species:Type, family = binomial)
Deviance Residuals:
    Min       1Q   Median       3Q      Max
-3.4168  -0.5924  -0.4540   1.1812   2.9188
Coefficients:
                  Estimate Std. Error z value Pr(>|z|)
(Intercept)         -4.1786     0.2148 -19.451  < 2e-16 ***
Species1        -2.4240     0.7395  -3.278 0.001045 **
Species2        -2.1068     0.6165  -3.417 0.000633 ***
```

```
Type1               8.8998     0.3518  25.298  < 2e-16 ***
Species1:Type1  -2.7653     0.7917  -3.493 0.000478 ***
Species2:Type1  -5.3622     0.6846  -7.833 4.78e-15 ***
---
Signif. codes:  0 '***' 0.001 '**' 0.01 '*' 0.05 '.' 0.1 ' ' 1
(Dispersion parameter for binomial family taken to be 1)
    Null deviance: 6995.17  on 95  degrees of freedom
Residual deviance:  148.04  on 90  degrees of freedom
AIC: 343.87
Number of Fisher Scoring iterations: 6


glm(formula = Dbin ~ Species + logTHSp1 + Type + Species:Type +
      Species:logTHSp1, family = binomial)
Deviance Residuals:
    Min       1Q   Median       3Q      Max
-2.9066  -0.6353  -0.4637   1.1636   2.9862
Coefficients:
                     Estimate Std. Error z value Pr(>|z|)
(Intercept)          -4.12126    0.36384 -11.327  < 2e-16 ***
Species1          -2.61915    0.80078  -3.271 0.001073 **
Species2          -1.81343    0.70012  -2.590 0.009593 **
logTHSp1             -0.01075    0.05560  -0.193 0.846637
Type1                 8.90092    0.35197  25.289  < 2e-16 ***
Species1:Type1    -2.76342    0.79175  -3.490 0.000483 ***
Species2:Type1    -5.35774    0.68475  -7.824  5.1e-15 ***
Species1:logTHSp1  0.03566    0.05786   0.616 0.537679
Species2:logTHSp1 -0.05794    0.06435  -0.900 0.367952
---
Signif. codes:  0 '***' 0.001 '**' 0.01 '*' 0.05 '.' 0.1 ' ' 1
(Dispersion parameter for binomial family taken to be 1)
    Null deviance: 6995.17  on 95  degrees of freedom
Residual deviance:  141.16  on 87  degrees of freedom
AIC: 342.99
Number of Fisher Scoring iterations: 6

glm(formula = Dbin ~ Species + logTHSp1 + Type + Size + Species:Type +
      Species:logTHSp1 + Species:Size + logTHSp1:Size, family = binomial)
Deviance Residuals:
    Min       1Q   Median       3Q      Max
-2.3977  -0.6811  -0.3089   0.5885   2.1149
Coefficients: (1 not defined because of singularities)
```

```
                     Estimate Std. Error z value Pr(>|z|)
(Intercept)           -5.3494     0.5568  -9.607  < 2e-16 ***
Species1           -1.6118     0.9089  -1.773  0.07617 .
Species2           -0.8858     0.8342  -1.062  0.28826
logTHSp1              -0.3888     0.2066  -1.882  0.05988 .
Type1                  9.4394     0.4344  21.730  < 2e-16 ***
Size3                  2.2006     1.0513   2.093  0.03633 *
Size30                 1.3035     1.4603   0.893  0.37205
Size300                4.0872     1.9208   2.128  0.03335 *
Species1:Type1     -3.2970     0.8316  -3.964 7.36e-05 ***
Species2:Type1     -5.8932     0.7307  -8.065 7.33e-16 ***
Species1:logTHSp1   0.3222     0.1984   1.624  0.10440
Species2:logTHSp1  -0.3269     0.2266  -1.443  0.14912
Species1:Size3     -2.7668     1.0700  -2.586  0.00972 **
Species2:Size3     -0.6664     1.1783  -0.566  0.57168
Species1:Size30    -2.2105     1.4325  -1.543  0.12281
Species2:Size30     1.6778     1.5999   1.049  0.29431
Species1:Size300   -3.2557     1.8585  -1.752  0.07981 .
Species2:Size300    1.5384     2.0744   0.742  0.45832
logTHSp1:Size3         0.3322     0.1175   2.828  0.00469 **
logTHSp1:Size30        0.2644     0.1201   2.201  0.02773 *
logTHSp1:Size300           NA         NA      NA       NA
---
Signif. codes:  0 ‘***’ 0.001 ‘**’ 0.01 ‘*’ 0.05 ‘.’ 0.1 ‘ ’ 1
(Dispersion parameter for binomial family taken to be 1)
    Null deviance: 6995.17  on 95  degrees of freedom
Residual deviance:   78.78  on 76  degrees of freedom
AIC: 302.61
Number of Fisher Scoring iterations: 6
```

```
glm(formula = Verandabin ~ Species + Type + Species:Type, family =
      binomial)
Deviance Residuals:
    Min       1Q   Median       3Q      Max
-6.3324  -1.3388   0.0852   0.9334   8.5091
Coefficients:
                  Estimate Std. Error z value Pr(>|z|)
(Intercept)         0.14842    0.05252   2.826  0.00472 **
Species1         0.16857    0.07441   2.265  0.02349 *
Species2        -1.28796    0.07832 -16.444  < 2e-16 ***
Type1               0.04636    0.07416   0.625  0.53194
```

```
Species1:Type1  0.50646    0.10430   4.856  1.2e-06 ***
Species2:Type1  1.58320    0.10729  14.756  < 2e-16 ***
---
Signif. codes:  0 ‘***’ 0.001 ‘**’ 0.01 ‘*’ 0.05 ‘.’ 0.1 ‘ ’ 1
(Dispersion parameter for binomial family taken to be 1)
    Null deviance: 1310.38  on 95  degrees of freedom
Residual deviance:  456.25  on 90  degrees of freedom
AIC: 931.41
Number of Fisher Scoring iterations: 4
```

```
glm(formula = Verandabin ~ Species + logTHSp1 + Type + Species:Type +
      Species:logTHSp1, family = binomial)

Deviance Residuals:
    Min       1Q   Median       3Q      Max
-6.3183  -1.1233   0.0976   0.9707   4.3893
Coefficients:
                      Estimate Std. Error z value Pr(>|z|)
(Intercept)           0.154966   0.083649   1.853   0.0639 .
Species1           0.305163   0.119094   2.562   0.0104 *
Species2          -0.527844   0.119890  -4.403 1.07e-05 ***
logTHSp1             -0.001217   0.012103  -0.101   0.9199
Type1                 0.046360   0.074162   0.625   0.5319
Species1:Type1     0.506418   0.104334   4.854 1.21e-06 ***
Species2:Type1     1.671083   0.109525  15.258  < 2e-16 ***
Species1:logTHSp1 -0.025108   0.017155  -1.464   0.1433
Species2:logTHSp1 -0.149586   0.017820  -8.394  < 2e-16 ***
---
Signif. codes:  0 ‘***’ 0.001 ‘**’ 0.01 ‘*’ 0.05 ‘.’ 0.1 ‘ ’ 1
(Dispersion parameter for binomial family taken to be 1)
    Null deviance: 1310.38  on 95  degrees of freedom
Residual deviance:  311.82  on 87  degrees of freedom
AIC: 792.99
Number of Fisher Scoring iterations: 4
```

```
glm(formula = Verandabin ~ Species + logTHSp1 + Type + Size + Species:Type
      + Species:logTHSp1 + Type:Size + logTHSp1:Size + Species:Size +
      Species:Type:Size, family = binomial)
Deviance Residuals:
    Min       1Q   Median       3Q      Max
```

```
-3.7845  -0.6197   0.0044   0.6343   3.3020
Coefficients: (1 not defined because of singularities)
                          Estimate Std. Error z value Pr(>|z|)
(Intercept)                0.16990    0.13066   1.300 0.193490
Species1                0.41918    0.18749   2.236 0.025366 *
Species2               -0.23734    0.17908  -1.325 0.185064
logTHSp1                   0.25193    0.05591   4.506 6.59e-06 ***
Type1                     -0.10588    0.18190  -0.582 0.560528
Size3                      0.29519    0.28733   1.027 0.304242
Size30                    -0.60067    0.38784  -1.549 0.121439
Size300                   -1.84946    0.51669  -3.579 0.000344 ***
Species1:Type1          0.48937    0.26325   1.859 0.063033 .
Species2:Type1          0.99683    0.25749   3.871 0.000108 ***
Species1:logTHSp1      -0.09567    0.05919  -1.616 0.106000
Species2:logTHSp1      -0.27851    0.06264  -4.446 8.73e-06 ***
Type1:Size3                0.86204    0.23272   3.704 0.000212 ***
Type1:Size30               0.28130    0.22788   1.234 0.217050
Type1:Size300             -0.62263    0.23468  -2.653 0.007975 **
logTHSp1:Size3            -0.36593    0.06109  -5.990 2.10e-09 ***
logTHSp1:Size30           -0.18817    0.06051  -3.110 0.001871 **
logTHSp1:Size300                NA         NA      NA       NA
Species1:Size3          0.26251    0.34235   0.767 0.443202
Species2:Size3          0.32706    0.34763   0.941 0.346794
Species1:Size30         0.55091    0.45040   1.223 0.221274
Species2:Size30         0.09978    0.47151   0.212 0.832401
Species1:Size300        0.09215    0.56982   0.162 0.871533
Species2:Size300        0.79419    0.59838   1.327 0.184433
Species1:Type1:Size3   -0.67107    0.32895  -2.040 0.041350 *
Species2:Type1:Size3    0.32855    0.33493   0.981 0.326618
Species1:Type1:Size30  -0.04961    0.32917  -0.151 0.880208
Species2:Type1:Size30   1.28746    0.33860   3.802 0.000143 ***
Species1:Type1:Size300  0.82008    0.33298   2.463 0.013785 *
Species2:Type1:Size300  1.01402    0.33222   3.052 0.002271 **
---
Signif. codes:  0 ‘***’ 0.001 ‘**’ 0.01 ‘*’ 0.05 ‘.’ 0.1 ‘ ’ 1
(Dispersion parameter for binomial family taken to be 1)
    Null deviance: 1310.38  on 95  degrees of freedom
Residual deviance:  148.58  on 67  degrees of freedom
AIC: 669.75
Number of Fisher Scoring iterations: 4
```

```
glm(formula = Netbin ~ Species + Type + Species:Type, family = binomial)
Deviance Residuals:
     Min        1Q    Median        3Q       Max
-16.4793   -1.8969    0.6938    1.9064    6.3286
Coefficients:
                  Estimate Std. Error z value Pr(>|z|)
(Intercept)       -1.83318    0.07596 -24.132  < 2e-16 ***
Species1       -0.49577    0.11894  -4.168 3.07e-05 ***
Species2        1.42203    0.09142  15.556  < 2e-16 ***
Type1              0.14081    0.10460   1.346    0.178
Species1:Type1 -0.73879    0.17472  -4.228 2.35e-05 ***
Species2:Type1 -1.23336    0.13304  -9.271  < 2e-16 ***
---
Signif. codes:  0 '***' 0.001 '**' 0.01 '*' 0.05 '.' 0.1 ' ' 1
(Dispersion parameter for binomial family taken to be 1)
    Null deviance: 2038.3  on 95  degrees of freedom
Residual deviance: 1227.7  on 90  degrees of freedom
AIC: 1590.9
Number of Fisher Scoring iterations: 5
```

```
glm(formula = Netbin ~ Species + logTHSp1 + Type + Species:Type +
      logTHSp1:Type + Species:logTHSp1 + Species:logTHSp1:Type, family =
      binomial)
Deviance Residuals:
    Min       1Q   Median       3Q      Max
-8.3607  -2.0840  -0.0518   1.4538   5.5201
Coefficients:
                           Estimate Std. Error z value Pr(>|z|)
(Intercept)                -2.77099    0.19732 -14.043  < 2e-16 ***
Species1                -1.08434    0.34337  -3.158 0.001589 **
Species2                 0.80383    0.23899   3.363 0.000770 ***
logTHSp1                    0.15942    0.02861   5.572 2.52e-08 ***
Type1                      -1.17241    0.32209  -3.640 0.000273 ***
Species1:Type1          -0.52411    0.59618  -0.879 0.379335
Species2:Type1          -0.50409    0.41455  -1.216 0.223985
logTHSp1:Type1              0.19280    0.04428   4.354 1.34e-05 ***
Species1:logTHSp1        0.08412    0.04748   1.772 0.076426 .
Species2:logTHSp1        0.11127    0.03511   3.169 0.001527 **
Species1:logTHSp1:Type1 -0.04361    0.07825  -0.557 0.577278
Species2:logTHSp1:Type1 -0.12771    0.05721  -2.232 0.025595 *
---
```

```
Signif. codes:  0 ‘***’ 0.001 ‘**’ 0.01 ‘*’ 0.05 ‘.’ 0.1 ‘ ’ 1
(Dispersion parameter for binomial family taken to be 1)
    Null deviance: 2038.28  on 95  degrees of freedom
Residual deviance:  532.09  on 84  degrees of freedom
AIC: 907.24
Number of Fisher Scoring iterations: 5


glm(formula = Netbin ~ Species + logTHSp1 + Type + Size + logTHSp1:Size +
      Species:Type + logTHSp1:Type + Species:logTHSp1 + Species:Size +
      Type:Size + Species:logTHSp1:Size + logTHSp1:Type:Size +
      Species:Type:Size, family = binomial)
Deviance Residuals:
    Min       1Q   Median       3Q      Max
-2.7666  -0.8382  -0.1476   0.6012   2.4342
Coefficients: (4 not defined because of singularities)
                               Estimate Std. Error z value Pr(>|z|)
(Intercept)                    -4.76217    0.71012  -6.706 2.00e-11 ***
Species1                    -0.02114    1.00422  -0.021  0.98320
Species2                   -17.52305 2563.13497  -0.007  0.99455
logTHSp1                       -0.22014    0.10905  -2.019  0.04351 *
Type1                         -17.45759 2563.55463  -0.007  0.99457
Size3                           0.63787    1.01587   0.628  0.53007
Size30                          3.10135    0.99153   3.128  0.00176 **
Size300                         4.98061    1.18872   4.190 2.79e-05 ***
logTHSp1:Size3                  0.71765    0.19011   3.775  0.00016 ***
logTHSp1:Size30                 0.26781    0.15060   1.778  0.07536 .
logTHSp1:Size300                     NA         NA      NA       NA
Species1:Type1              -0.08091 3624.95946   0.000  0.99998
Species2:Type1              34.18985 3625.11707   0.009  0.99247
logTHSp1:Type1                 -0.09205    0.09877  -0.932  0.35135
Species1:logTHSp1           -0.06456    0.14079  -0.459  0.64654
Species2:logTHSp1            0.22038    0.11484   1.919  0.05497 .
Species1:Size3              -4.74850    2.00816  -2.365  0.01805 *
Species2:Size3              18.94224 2563.13511   0.007  0.99410
Species1:Size30             -6.62859    1.65566  -4.004 6.24e-05 ***
Species2:Size30             18.52808 2563.13509   0.007  0.99423
Species1:Size300             0.37130    1.58727   0.234  0.81504
Species2:Size300            17.16130 2563.13517   0.007  0.99466
Type1:Size3                    13.75643 2563.55485   0.005  0.99572
Type1:Size30                   15.34029 2563.55475   0.006  0.99523
Type1:Size300                  19.06343 2563.55478   0.007  0.99407
```

```
Species1:logTHSp1:Size3      0.92348    0.37875   2.438  0.01476 *
Species2:logTHSp1:Size3     -0.21112    0.21339  -0.989  0.32248
Species1:logTHSp1:Size30     0.94240    0.23459   4.017 5.89e-05 ***
Species2:logTHSp1:Size30    -0.10696    0.16482  -0.649  0.51637
Species1:logTHSp1:Size300         NA         NA      NA       NA
Species2:logTHSp1:Size300         NA         NA      NA       NA
logTHSp1:Type1:Size3            0.74329    0.23162   3.209  0.00133 **
logTHSp1:Type1:Size30           0.40905    0.14924   2.741  0.00613 **
logTHSp1:Type1:Size300               NA         NA      NA       NA
Species1:Type1:Size3        -1.81884 3624.95951  -0.001  0.99960
Species2:Type1:Size3       -35.26199 3625.11708  -0.010  0.99224
Species1:Type1:Size30       -0.53455 3624.95947   0.000  0.99988
Species2:Type1:Size30      -35.82615 3625.11708  -0.010  0.99211
Species1:Type1:Size300      -0.99175 3624.95947   0.000  0.99978
Species2:Type1:Size300     -35.61632 3625.11708  -0.010  0.99216
---
Signif. codes:  0 ‘***’ 0.001 ‘**’ 0.01 ‘*’ 0.05 ‘.’ 0.1 ‘ ’ 1
(Dispersion parameter for binomial family taken to be 1)
    Null deviance: 2038.28  on 95  degrees of freedom
Residual deviance:  112.76  on 60  degrees of freedom
AIC: 535.92
Number of Fisher Scoring iterations: 16
```
