# Supplementary material for: Do holes in long-lasting insecticidal nets compromise their efficacy against pyrethroid resistant Anopheles gambiae and Culex quinquefasciatus? Results from a release–recapture study in experimental huts
Source: Malar J. 2015 Aug 28;14:332. doi: 10.1186/s12936-015-0836-7 (PMC4551388; doi:10.1186/s12936-015-0836-7)
Supplement: Additional file 5: — Figures for population effects against feeding, community insecticidal effectiveness and population effect on transmission depending on percent net use. [file 12936_2015_836_MOESM5_ESM.pdf]

Additional file 5. Figures for population effects against feeding, community insecticidal effectiveness and population effect on transmission depending on percent net use.

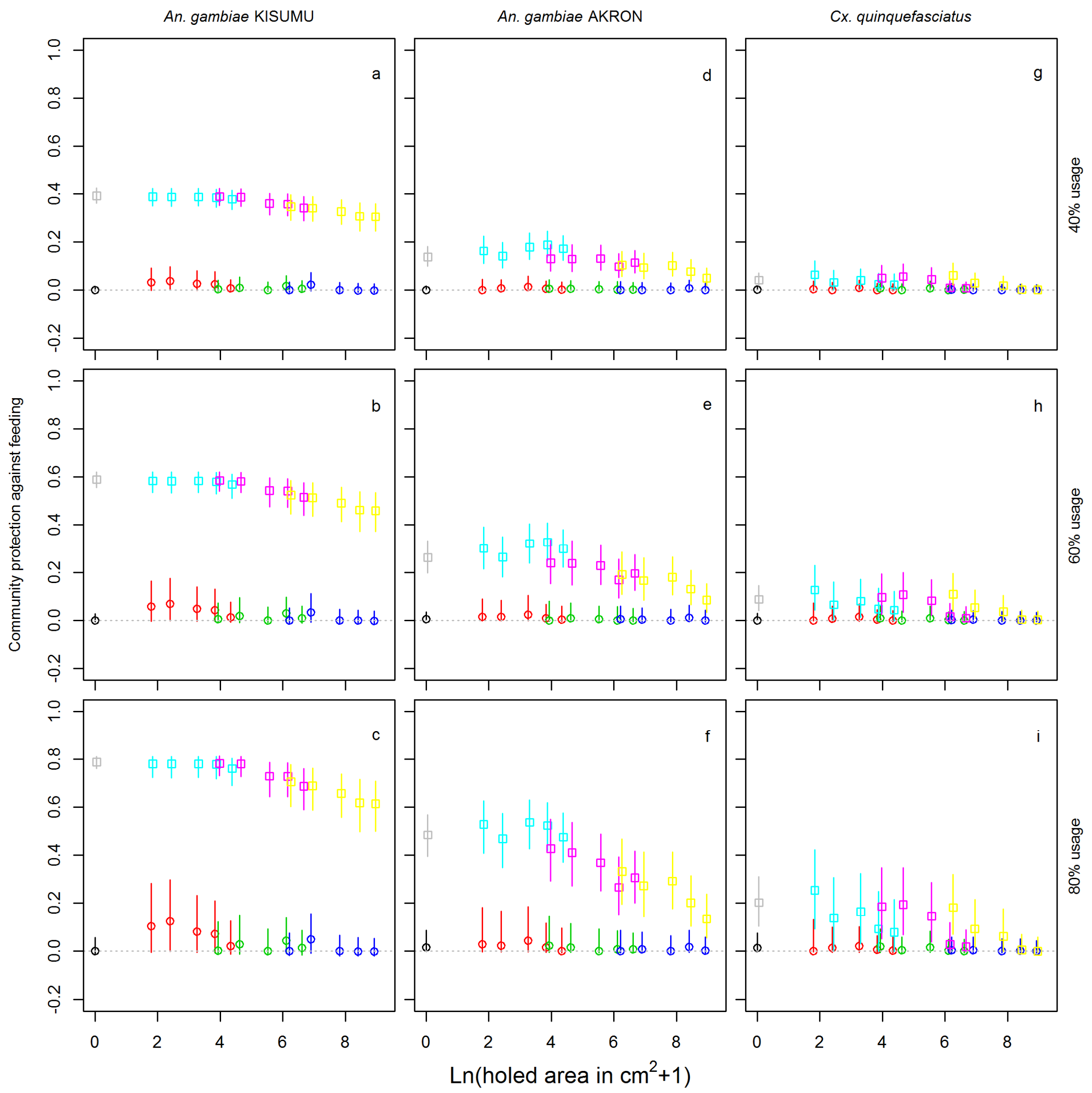

Figure A5.1 Community protection against feeding, depending on net usage in the population. Legend: See Figure 4.

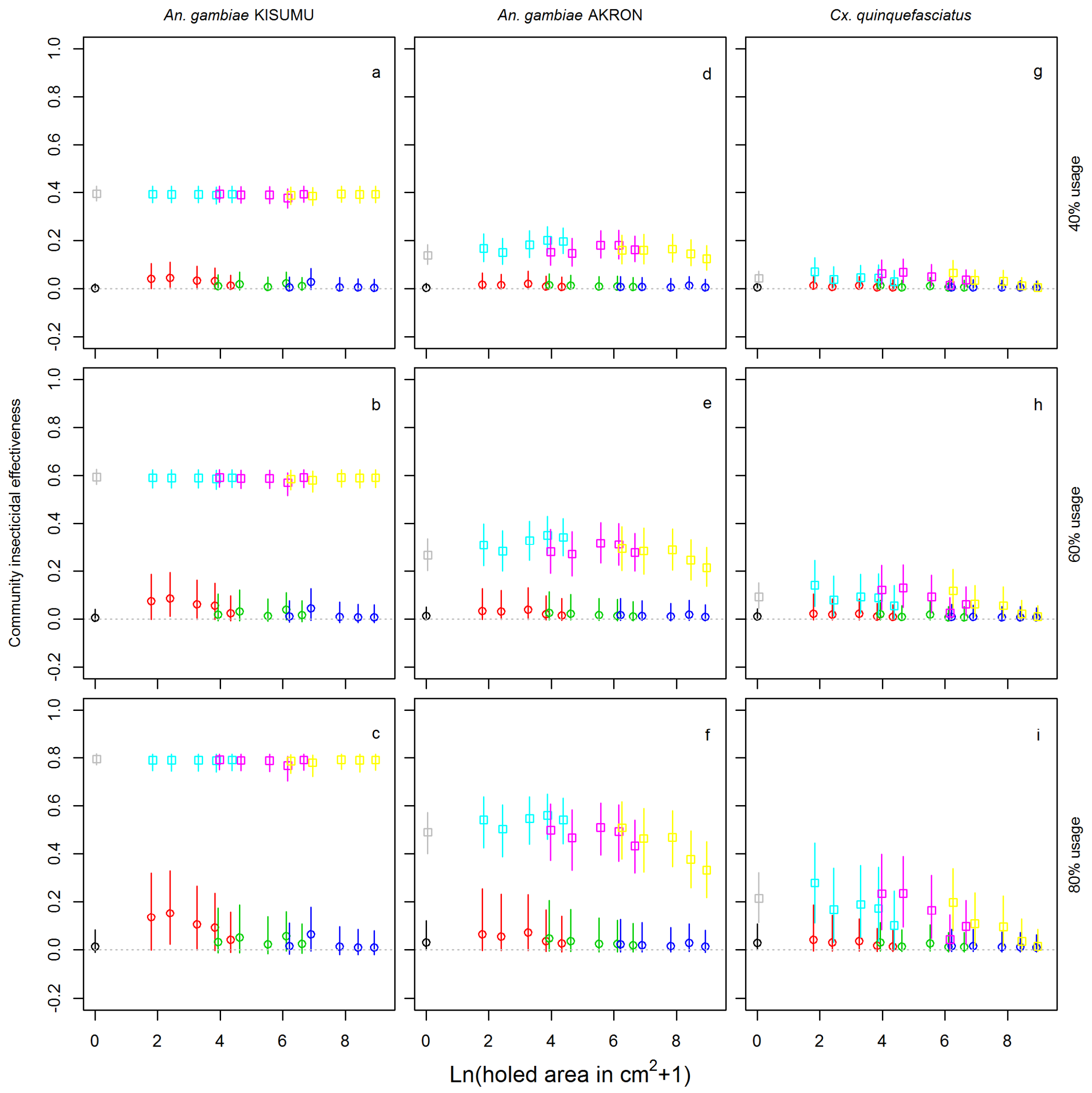

Figure A5.2 Community insecticidal effectiveness, depending on net usage in the population. Legend: See Figure 4.

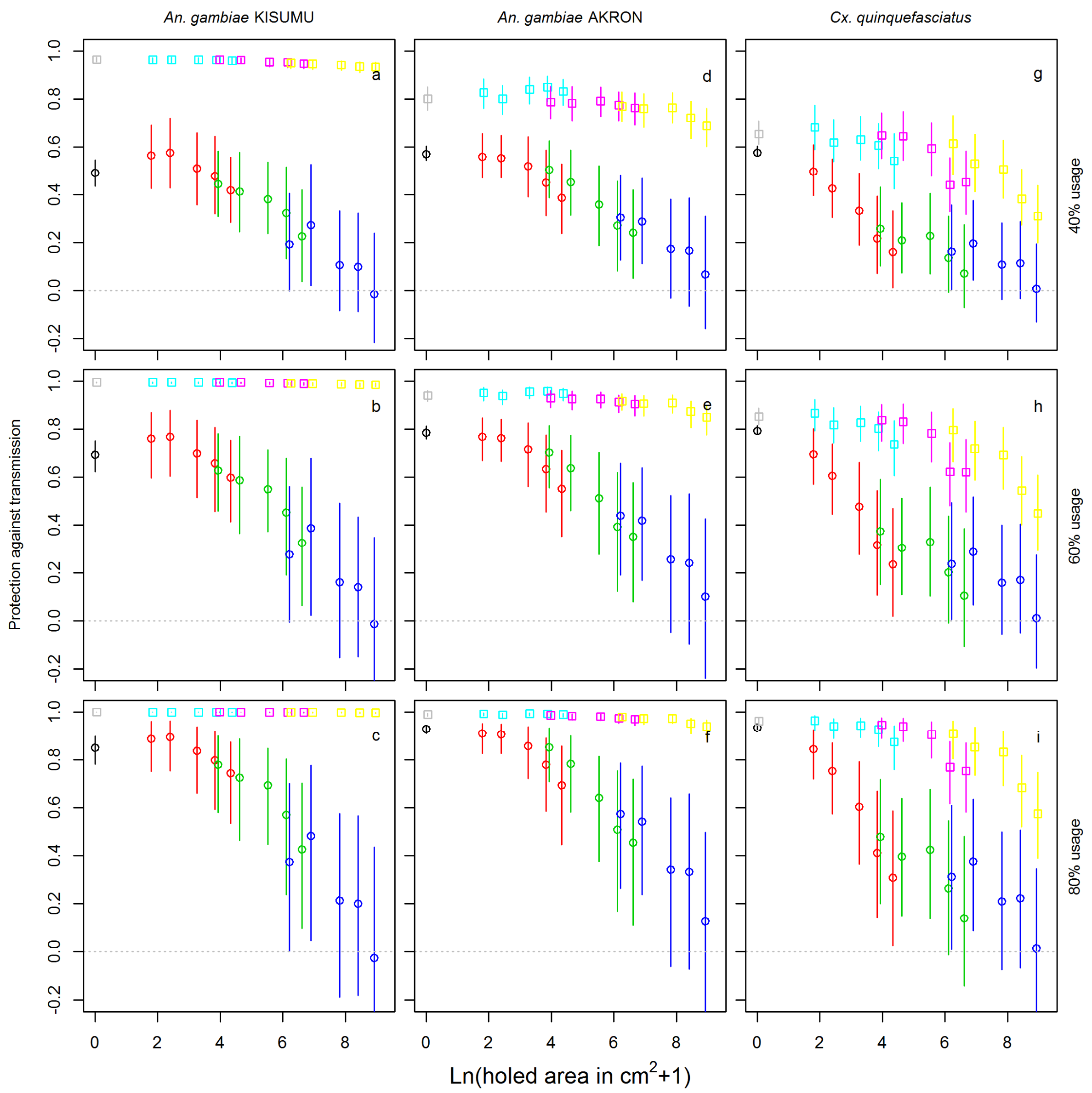

Figure A5.3 Protection against transmission, depending on net usage in the population. Legend: See Figure 4.
