# Supplementary material for: Do holes in long-lasting insecticidal nets compromise their efficacy against pyrethroid resistant Anopheles gambiae and Culex quinquefasciatus? Results from a release–recapture study in experimental huts
Source: Malar J. 2015 Aug 28;14:332. doi: 10.1186/s12936-015-0836-7 (PMC4551388; doi:10.1186/s12936-015-0836-7)
Supplement: Additional file 6: — Risk ratios of mosquitoes being dead depending on being fed or unfed. [file 12936_2015_836_MOESM6_ESM.pdf]

Additional file 6. Risk ratios of mosquitoes being dead depending on being fed or unfed.

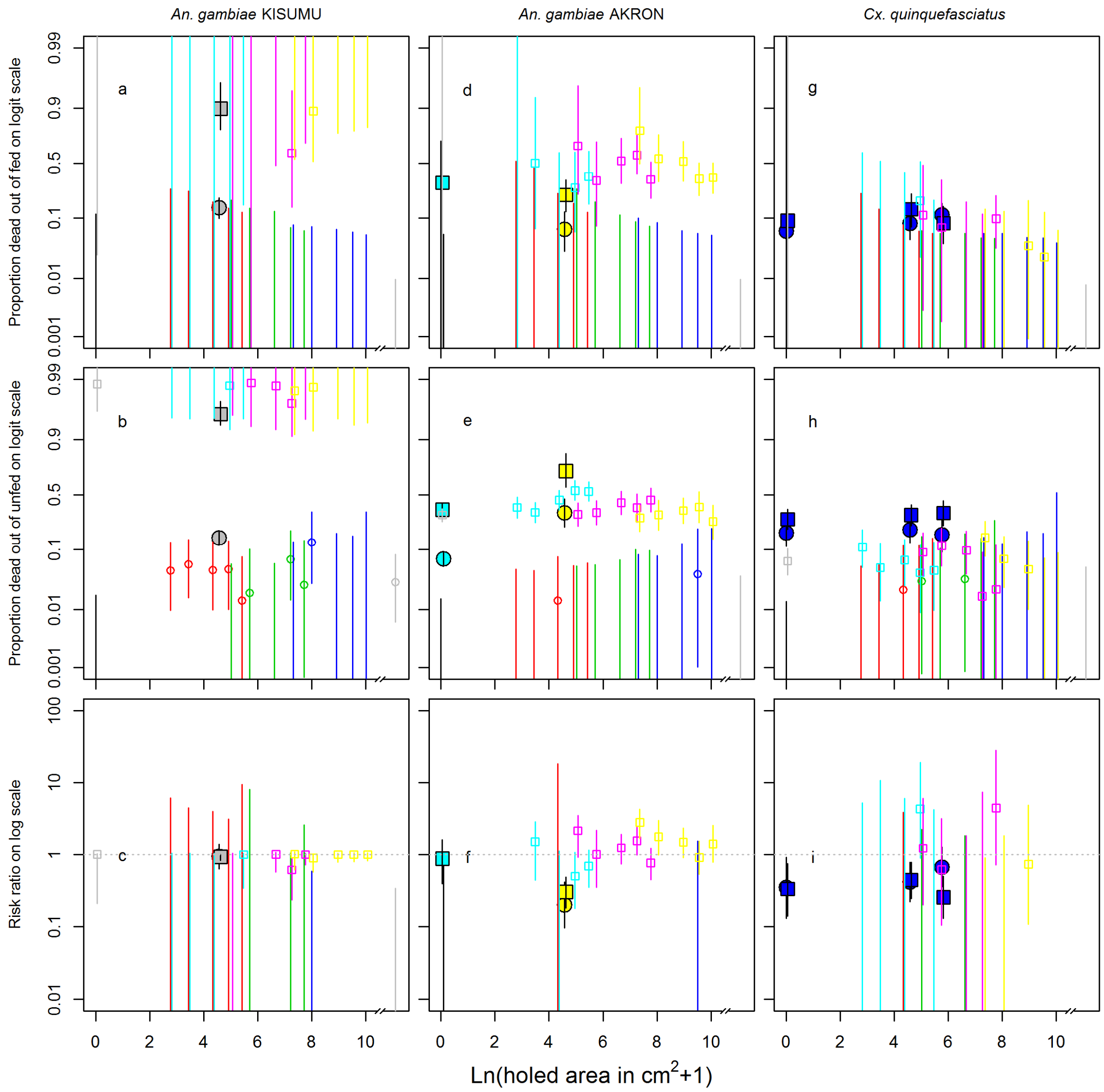

Risk ratio of mosquitoes being dead depending on having fed or not and on the holed surface area on three mosquito species in relation to data in literature. The first column of panels (**a** – **c**) is for *An. gambiae* KISUMU strain, the second column of panels (**d** – **f**) is for *An. gambiae* collected in Akron, and the third row of panels (**g** – **i**) is for *Cx. quinquefasciatus* collected in Cotonou. The first row of panels (**a**, **d**, **g**) shows the proportion dead out of fed mosquitoes, the second row of panels (**b**, **e**, **h**) shows the proportion dead out of unfed and the third row of panels (**c**, **f**, **i**) shows the risk ratio of mosquitoes being dead depending on having fed or not. Symbols show mean values with grey squares representing intact LLINs; cyan squares LLINs with holes of 3 cm^2; magenta squares LLINs with holes of 30 cm^2; yellow squares LLINs with holes of 300 cm^2; black circles intact untreated nets; red circles untreated nets with holes of 3 cm^2; lime green circles untreated nets with holes of 30 cm^2; dark blue circles untreated nets with holes of 300 cm^2. Grey circles represent the results from huts

without nets. Vertical lines show 95% confidence intervals, and grey lines show the fitted relationships from logistic regressions with net type, mosquito species and holed surface area as explanatory variables. Larger symbols with black outlines represent data from literature with fill colour grey from Tungu and colleagues [27], fill colour yellow from N'Guessan and colleagues [29], fill colour cyan from Koudou and colleagues [60], and fill colour dark blue from Irish and colleagues [35].
